# Boosting BCG-primed responses with a subunit Apa vaccine during the waning phase improves immunity and imparts protection against *Mycobacterium tuberculosis*

**DOI:** 10.1038/srep25837

**Published:** 2016-05-13

**Authors:** Subhadra Nandakumar, Sunil Kannanganat, Karen M. Dobos, Megan Lucas, John S. Spencer, Rama Rao Amara, Bonnie B. Plikaytis, James E. Posey, Suraj B. Sable

**Affiliations:** 1 Division of Tuberculosis Elimination, National Center for HIV/AIDS, Viral Hepatitis, STD, and TB Prevention, Centers for Disease Control and Prevention, Atlanta, GA 30333, USA; 2 Department of Microbiology and Immunology, Emory Vaccine Center and Yerkes National Primate Research Center, Emory University, Atlanta, GA 30329, USA; 3 Department of Microbiology, Immunology and Pathology, College of Veterinary Medicine and Biomedical Sciences, Colorado State University, Fort Collins, CO 80523, USA

## Abstract

Heterologous prime–boosting has emerged as a powerful vaccination approach against tuberculosis. However, optimal timing to boost BCG-immunity using subunit vaccines remains unclear in clinical trials. Here, we followed the adhesin Apa-specific T-cell responses in BCG-primed mice and investigated its BCG-booster potential. The Apa-specific T-cell response peaked 32–52 weeks after parenteral or mucosal BCG-priming but waned significantly by 78 weeks. A subunit-Apa-boost during the contraction-phase of BCG-response had a greater effect on the magnitude and functional quality of specific cellular and humoral responses compared to a boost at the peak of BCG-response. The cellular response increased following mucosal BCG-prime–Apa-subunit-boost strategy compared to Apa-subunit-prime–BCG-boost approach. However, parenteral BCG-prime–Apa-subunit-boost by a homologous route was the most effective strategy in-terms of enhancing specific T-cell responses during waning in the lung and spleen. Two Apa-boosters markedly improved waning BCG-immunity and significantly reduced *Mycobacterium tuberculosis* burdens post-challenge. Our results highlight the challenges of optimization of prime–boost regimens in mice where BCG drives persistent immune-activation and suggest that boosting with a heterologous vaccine may be ideal once the specific persisting effector responses are contracted. Our results have important implications for design of prime–boost regimens against tuberculosis in humans.

Improved vaccination approaches against tuberculosis (TB) are urgently needed. The only vaccine currently licensed against TB, Bacille Calmette-Guérin (BCG), affords highly variable and inadequate protection against pulmonary TB^1^. Yet, BCG remains the most widely administered human vaccine in the world, partly due to its ability to protect against severe and extrapulmonary forms of TB in children^2^. The protection afforded by BCG is thought to wane with time after vaccination at birth and is generally considered to last only through adolescent years^3^, although protection surpassing 5–6 decades has been reported in some populations^4^.

Currently, one of the reigning strategies in TB vaccine research is to develop BCG-booster vaccines using adjuvanted protein-subunit or viral-vectored vaccines^5^. These heterologous prime–boost strategies have proven a powerful mode of vaccination. However, optimization of these heterologous prime–boost regimens has been impeded by incomplete understanding of the dynamics of BCG-elicited responses in human and animal hosts. Furthermore, the optimal timing to boost BCG-primed immunity remains uncertain. The mouse model has been extensively used to evaluate new prime–boost strategies using BCG. However, most published studies using investigational vaccines have employed an empirical approach to boost BCG-immunity^5^. It has become increasingly clear that BCG elicits fairly enduring CD4^+^ and CD8^+^ T-cell responses in mice and humans with varying expansion versus contraction-phase-specific functional characteristics^6^,^7^,^8^. The protein-subunit vaccines, which are often investigated for their BCG-boosting potential, also prime relatively durable immune responses^9^. However, influence of such persisting T-cell responses primed by the live BCG vaccine on the boosting outcome of adjuvanted protein-subunit vaccines has not been systematically studied.

The expression of mycobacterial antigens is known to vary *in vivo* depending on the stage of bacillary growth^10^. Therefore, different antigens may exhibit diverse response kinetics in BCG-primed hosts and could potentially afford divergent boosting outcomes depending upon the timing of boost. Since boosting T-cell responses during the expansion-phase may lead to activation-induced cell death or T-cell exhaustion, boosting prior responses after the effector-phase when the memory T-cell population is established may be optimal^7^,^11^,^12^. However, the immunological outcomes of boosting during the expansion or peak-phase, when the immune responses are still robust, versus during the contraction-phase of BCG-immunity when the responses wane, have not been systematically studied. Another unresolved question is whether the homologous route of prime–boost vaccinations targeting same draining lymph nodes (LNs) provides a more effective boost than the heterologous routes of vaccinations targeting different/distant LNs, as compartmentalization and anatomical localization of T-cell responses differ between mucosal versus parenteral route of BCG-priming^8^,^13^. In this study, we used the mouse model to investigate the boosting potential of a protein-subunit vaccine using *Mycobacterium tuberculosis* (*Mtb*) alanine-proline-rich antigen (Apa) as a candidate booster antigen, when administered at the peak versus at the waning of specific BCG-primed response and by altering routes of prime–boost vaccinations.

Apa is a cell-surface adhesin and secretory glycoprotein produced by all members of *Mtb*-complex, including BCG^14^,^15^,^16^. The native Apa (nApa) is glycosylated and is preferentially recognized over non-glycosylated recombinant Apa (rApa) in BCG-vaccinated or *Mtb*-infected hosts, while both forms are immunogenic and impart notable protection as a subunit vaccine^17^. In multiple studies, Apa-subunit or DNA vaccines have been shown to impart significant protection against *Mtb* challenge, irrespective of the vaccination strategy or animal model used^13^,^17^,^18^,^19^. In this study, using BCG and subunit Apa vaccine, we demonstrate that the time-interval between prime and boost, the magnitude and quality of specific T-cell response persisting at the time of boost, and the route and sequence of primer and booster vaccine greatly influence the boosting outcome.

## Results

### Longitudinal changes in the magnitude and functional quality of the adhesin Apa-specific T cells in mucosal or parenteral BCG-primed mice

**Figure 1:**
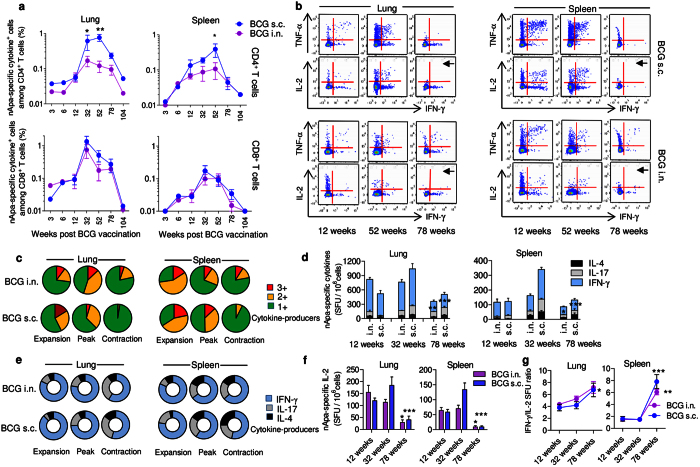
Longitudinal changes in *M. tuberculosis* Apa-specific T-cell cytokine responses in mucosal or parenteral BCG-vaccinated mice during the two-year life-span. BALB/c mice were vaccinated with 10^6^ CFU BCG Copenhagen by subcutaneous (s.c.) or intranasal (i.n.) route. At different time points after vaccination, as indicated, mice were euthanized (n = 4 mice/time point/group) and their lung and spleen cells (pools) were stimulated *in vitro* with or without nApa. (**a**) Frequencies of IFN-γ, IL-2 and/or TNF-α-producing cells among CD4^+^ or CD8^+^ T cells were determined using polychromatic flow cytometry and percentages (%) of nApa-specific total cytokine-producing cells among CD4^+^ and CD8^+^ T cells are plotted. Data at week 12, 32, 52 and 78 are means ± s.e.m. of 3–4 independent mouse experiments and data (means) at week 3, 6 and 104 are from one experiment evaluated in duplicate. (**b**) Representative flow-cytometric plots from one experiment showing changes in the cytokine co-expression profile of nApa-specific CD4^+^ T cells at week 12, 52 and 78. Arrows highlight the decline in the frequency of IL-2^+^CD4^+^ T cells at week 78. (**c**) Changes in the proportions of single (1+), double (2+) and triple (3+) cytokine-producing subsets constituting nApa-specific total cytokine^+^CD4^+^ T cells at the expansion (week12), peak (week 52) and contraction (week 78) phase of BCG-response in a. Pie charts present mean frequencies of 1+, 2+ or 3+ cytokine-producers. (**d**) Frequencies of nApa-specific IFN-γ, IL-17 or IL-4-producing cells at week 12, 32 and 78 in a cultured ELISPOT assay. (**e**) Changes in the proportions of IFN-γ, IL-17 and IL-4 spot forming units (SFU) constituting the total ELISPOT response in d. Pie charts present mean IFN-γ, IL-17 or IL-4 SFU/10^6^ cells. (**f**) nApa-specific IL-2 SFU/10^6^ lung or spleen cells and (**g**) kinetic changes in the ratio of IFN-γ/IL-2 SFU at week 12, 32 and 78. Data in d–g are means ± s.e.m. of 2 independent experiments evaluated in triplicate. Significance denotes decreased SFU (**d,f**) or increased ratio (**g**) compared to respective 32-week time point. *P < 0.05; **P < 0.01; ***P < 0.001 using one-way ANOVA followed by Bonferroni correction in a, d–g.

Mucosal prime-boost regimens targeting respiratory mucosa can evoke powerful lung-resident T cells and afford superior protection compared to parenteral vaccination^20^,^21^,^22^, and the route of vaccination can influence the type of T-cell response elicited^23^,^24^. We compared longitudinal changes in candidate nApa-specific T cells in intranasal (i.n.) and subcutaneous (s.c.) BCG-vaccinated mice. We used a flow cytometry to quantify CD4^+^ and CD8^+^ T cells expressing IFN-γ, TNF-α and/or IL-2 in the lung and spleen. While *Mtb* whole cell lysate (WCL) and PPD-specific T cells peak around 12–32 weeks^8^,^20^, nApa-specific total CD4^+^ T-cell response peaked gradually between 32–52 weeks (Fig. 1a), before waning until 104 weeks, irrespective of the route of BCG-priming. The magnitudes of specific CD4^+^ T cells were about 3–5-fold higher in the s.c. compared to the i.n. group at the peak of response. Interestingly, the peak CD4^+^ response elicited by s.c. BCG underwent a more marked contraction by week 78 (3.2–8.7-fold) compared to the peak response after i.n. BCG (1.7–2.6-fold waning). When we analyzed frequencies of nApa-specific IFN-γ, IL-2, or TNF-α-expressing CD4^+^ T cells individually, similar kinetic trends were observed (Supplementary Fig. S1a). The CD8^+^ T cells mainly produced IFN-γ (Supplementary Fig. S1b), and exhibited similar expansion and contraction trends in the two groups (Fig. 1a). These results demonstrate that nApa-specific T-cell responses elicited by mucosal or parenteral BCG-priming persist over 1 year and exhibit comparable kinetic patterns in mice.

Next, we analyzed the temporal changes in functional quality of nApa-specific CD4^+^ T-cell response by flow cytometry and found markedly decreased polyfunctional cytokine co-expression with almost complete disappearance of IL-2-producing cells at week 78 (contraction-phase) compared to weeks 12 (expansion-phase) and 52 (the peak of response) (Fig. 1b,c). We found an IFN-γ-dominated nApa-specific response during the three phases using a cultured ELISPOT assay (Fig. 1d), and contrary to other bacteria, we did not observe any preponderance of type-17 over type-1 response following i.n. BCG-priming^24^. However, an approximately 2-fold contraction in peak IFN-γ spot-forming-units (SFU) was found by week 78 (Fig. 1d), resulting in increased proportions of IL-17 and IL-4 SFU during the contraction-phase (Fig. 1e), and the frequency of IL-2 SFU, a cytokine associated with T-cell central memory (T_CM_) and proliferative potential^25^, also declined significantly (*P* < 0.05–0.001) by week 78 (Fig. 1f), confirming our flow-cytometry results. Simultaneously, specific IFN-γ/IL-2 SFU ratios increased during waning (Fig. 1g), which suggest a prolonged persistence of Apa/bacillary-load in BCG-primed mice^26^. Likewise when the memory phenotype was investigated, WCL-specific CD4^+^ and CD8^+^ T cells predominantly expressed CD44 but demonstrated weak CD62L or CCR-7 expression phenotype^8^, indicating perpetuation of activated effector/effector memory (T_EM_) response coincident with chronic persistence of BCG in mice^8^,^27^.

Together these results suggest that the ratios of specific subpopulations of nApa-specific CD4^+^ T cells differ between expansion, peak and contraction-phase of BCG-response and the functional quality of specific CD4^+^ T cells, in particular cytokine expression, undergoes substantial alterations during the contraction-phase, regardless of the route of BCG-priming.

### Timing of the adjuvanted-Apa-boost after BCG-priming influences the strength and functionality of the booster response

**Figure 2:**
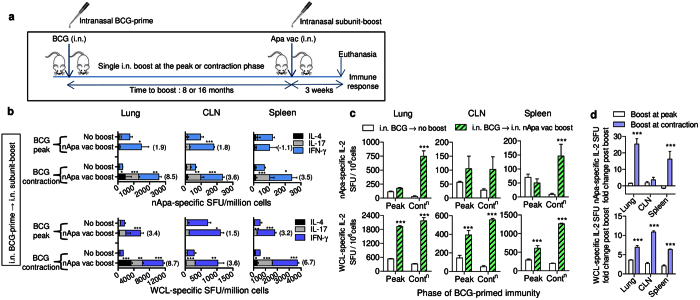
Boosting BCG-primed responses with a subunit nApa vaccine during the contraction-phase imparts a stronger booster effect than the boosting at the peak of response. (**a**) Schematic representation of the homologous route prime–boost vaccination protocol. Six–eight-week-old mice were primed i.n. with 10^6^ CFU BCG. Eight or sixteen months later, groups of mice received a single nApa (10 μg)-boost using a DDA-MPL adjuvant by a homologous i.n. route at the peak or contraction-phase of BCG-response. Three weeks following a subunit-boost, mice (n = 4/group/time point) were sacrificed and the magnitude and quality of cytokine response was investigated in the lung, CLN and spleen (pooled). (**b–d**) Cytokine responses using a cultured ELISPOT assay. Frequencies of nApa- (upper panels) or WCL-specific (lower panels) IFN-γ, IL-17 and IL-4 SFU (as a representative of type-1, type-17 and type-2 cytokine response) (**b**) and IL-2 SFU (a representative cytokine associated with proliferative, memory and/or regulatory potential) (**c**) per million organ cells are plotted for the nApa vaccine-boosted groups i.e., boosted at the BCG peak (8 months) or BCG contraction-phase (16 months) in comparison with the corresponding BCG-primed non-boosted controls. Data (in **b** and **c**) are means + s.d. of triplicate cultures. *P < 0.05; **P < 0.01 and ***P < 0.001 using a one-way ANOVA and Bonferroni correction and comparing the individual cytokine response. A number in the parenthesis (in **b**) indicates a fold change in the total IFN-γ, IL-17 and IL-4 SFU in the nApa-boosted mice over the corresponding non-boosted controls. A bar in (**d**) indicates a fold change in the specific IL-2 SFU (+ s.d.). Significance was derived by comparing fold-change between the two time-intervals. i.n., intranasal; vac, vaccine; WCL, whole cell lysate of *Mtb*; SFU, spot forming units; Cont^n^, contraction-phase.

To analyze the ability of subunit Apa to boost BCG-immunity and to understand the impact of time-interval between BCG-prime and protein-subunit-boost, we administered a single nApa (10 μg)-booster at the peak (8 months) or waning (16 months) of mucosal BCG-elicited response by a homologous i.n. route (Fig. 2a). The frequencies of immunogen (nApa) or mycobacteria (WCL)-specific IFN-γ, IL-17 or IL-4-producing cells were enumerated in the lung, spleen and draining cervical lymph nodes (CLN) using an ELISPOT assay at three weeks following a subunit-boost.

Compared to corresponding non-boosted controls, nApa-booster significantly increased nApa- and WCL-specific total SFU in three organs of BCG-primed mice (Fig. 2b, upper and lower panels), following administration at the peak or contraction-phase of BCG-response, except nApa-specific splenic booster response at the peak of BCG-immunity (Fig. 2b, upper right panel), where no significant increase was observed. Interestingly, total SFU increased 3.5–8.7-fold when the nApa-booster was administered after a prolonged interval during the contraction-phase (i.e., 16 months after BCG-priming) compared to the 1.5–3.4-fold increase after boosting at the peak of response (i.e., 8 months after BCG-priming) (Fig. 2b). Similar boosting dynamics were observed when the experiment was performed using rApa-booster (Supplementary Fig. S2a). These results suggest that the protein-subunit-boost offers a greater-fold increase in antigen-specific cellular responses when administered during the contraction-phase of BCG-response versus at the peak of effector response.

Strikingly, the ELISPOT response elicited by boosting during the contraction-phase consisted of significantly higher frequencies and proportions of specific IL-17 and IL-4 SFU (Fig. 2b); although a dominant and comparable IFN-γ booster response was found at both time intervals. The nApa-specific IL-2 response was significantly greater (*P* < 0.001) in the lung and spleen of contraction-phase-boosted group (Fig. 2c), with 15–25-fold boost over the corresponding non-boosted controls and 2.9–4.3-fold increase over the peak-boosted group (Fig. 2d). These results suggest that the timing of protein-subunit-boost after BCG-priming significantly influences the magnitude and quality of specific cellular response and indicate that the quality of persisting cytokine milieu at the time of boost likely determines the characteristics of subsequent booster response.

### The level of persisting BCG-primed immunity and its anatomical location impact the magnitude and distribution of a subsequent subunit-Apa-booster response

**Figure 3:**
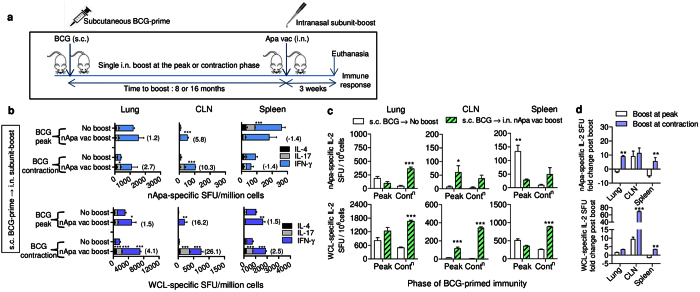
The level of persisting BCG-primed immunity and the route of nApa subunit-boost influence the magnitude and location of the cellular booster response. (**a**) Schematic representation of the heterologous route prime–boost vaccination protocol. Mice were primed s.c. with 10^6^ CFU BCG vaccine. Eight or sixteen months later, groups of mice received a single nApa (10 μg)-boost in a DDA/MPL adjuvant by the heterologous i.n. route at the peak or contraction-phase of BCG-response. Three weeks following a boost, mice (n = 4 mice/group/time point) were sacrificed and the magnitude and quality of the cellular immune response was investigated in the lung, CLN and spleen (pooled). (**b–d**) Cytokine response using a cultured ELISPOT assay. The frequencies of nApa-specific (upper panels) or WCL-specific (lower panels) IFN-γ, IL-17 and IL-4 SFU (**b**), or IL-2 SFU (**c**) per million cells are plotted for the nApa-boosted groups i.e., boosted at the BCG peak (8 months) or BCG contraction-phase (16 months) in comparison with the respective BCG-primed non-boosted controls. Data (in **b**,**c**) represent means + s.d. of triplicate cultures. *P < 0.05; **P < 0.01 and ***P < 0.001 using a one-way ANOVA and Bonferroni correction and comparing the individual cytokine response. A number in the parenthesis (in **b**) indicates a fold change in the total IFN-γ, IL-17 and IL-4 SFU in the nApa-boosted mice over the corresponding non-boosted controls. A bar in (**d**) indicates a fold change in the specific IL-2 SFU (+ s.d.). Significance was derived by comparing a fold-change at the two time intervals. s.c., subcutaneous; i.n., intranasal; vac, vaccine; WCL, whole cell lysate of *Mtb*; SFU, spot forming units; Cont^n^, contraction-phase.

Parenteral vaccination via skin is used to vaccinate infants with BCG, and the experimental evidence suggests that although parenteral BCG induces robust systemic responses, it elicits poor local resident T-cell responses^20^,^28^. The ‘prime and pull’ vaccination approach employing mucosal application of stimuli can pull systemic/circulating T-cell responses primed by parenteral vaccination in the local anatomical compartments and impart a significant tissue-resident cellular boost with improved protection^29^. Thus, we investigated the ability of a single i.n. nApa or rApa (10 μg)-booster to enhance s.c. BCG-primed responses (Fig. 3a) in the local and peripheral compartments 3 weeks post-boosting.

We found that a nApa-boost by heterologous i.n. route significantly increased nApa- or WCL-specific IFN-γ, IL-17 or IL-2 SFU in local CLNs, which drain the nasal cavity and upper respiratory tract (Fig. 3b,c), regardless of whether it was administered at the peak (8 months) or contraction-phase (16 months) of BCG-response. Conversely, no significant increase in nApa-specific SFU was found in the lung or spleen following a boost at the peak of s.c. BCG-response, when specific cytokine-producing cells preexisted in strong numbers in these two organs (Fig. 3b). In fact, the frequency of nApa-specific SFU in the spleen decreased after a subunit-boost at the peak, suggesting a possible ‘pull’ from the systemic compartment to local anatomical sites. Nevertheless, a greater-fold boost in WCL-specific SFU was found when a 16-month boosting-interval was employed (Fig. 3b,d). These results collectively suggest that the amount of persisting BCG-primed immunity and its anatomical location at the time of boost influence the magnitude and distribution of subsequent cellular booster response contingent upon the route of boosting. In summary, we conclude that the temporal and spatial differences in preexisting BCG-primed immunity strikingly impact the subunit vaccine-induced booster response.

Similar dynamics were observed when the experiment was performed using rApa-booster (Supplementary Fig. S2b). However, unlike the specific IFN-γ, IL-4 and IL-17 response, a significantly higher frequency of IL-2 SFU was found in nApa- compared to rApa-boosted mice (Supplementary Fig. S2c,d). These results allude to the potential impact of Apa-glycosylation on booster IL-2 response in BCG-primed mice.

We also considered the possibility that mucosal application of DDA-MPL stimuli might increase a cellular-influx into the lungs. We found that the i.n. administration of adjuvant alone in i.n. BCG-primed mice during the contraction-phase was able to increase IFN-γ-producing cells in the lung (1757±61 SFU/million cells) 1 week post-boosting independent of *in vitro* antigen-stimulation but not in the CLN or spleen. This spontaneous, nonspecific IFN-γ response was transient, and no significant difference in nApa-specific IFN-γ, IL-4 and IL-17 response was observed in the three organs compared to non-boosted BCG-primed controls at 3 weeks post adjuvant-boost (Supplementary Fig. S2e). Furthermore, the 1.4–1.7-fold increase observed in WCL-specific response following adjuvant-boost was also markedly lower than the 3.6–10.1-fold increase recorded after subunit Apa vaccine-boost (Supplementary Fig. S2a,e and Fig. 2b). These results suggest that the adjuvant-boost alone is not sufficient to increase durable responses and administration of antigen (specific stimuli) together with adjuvant (inflammatory stimuli) is required to effectively pull and maintain enduring antigen-specific cellular response.

### Boosting subunit Apa vaccine-primed responses with BCG does not afford a stronger boost

**Figure 4:**
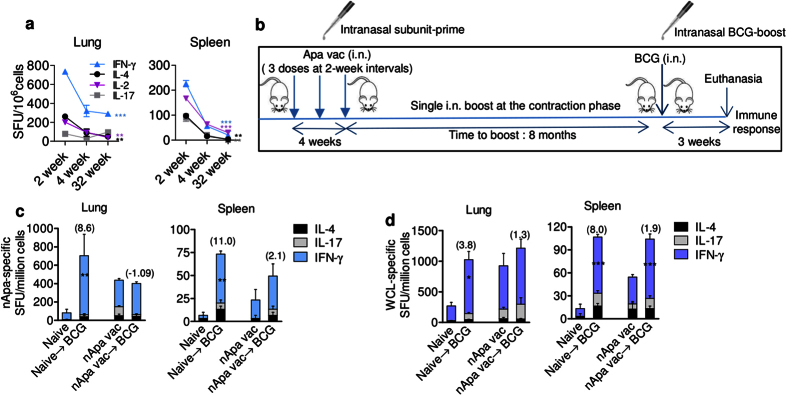
Boosting with BCG in the nApa subunit vaccine-primed mice does not impart a stronger booster effect. (**a**) The magnitude and kinetics of cytokine-producing cells in the i.n. mucosal nApa vaccinated mice. Mice were immunized three times with nApa (10 μg/dose) in a DDA-MPL adjuvant at 2-week intervals by the i.n. route, and the nApa-specific IFN-γ, IL-2, IL-4 and IL-17 responses were investigated in the lung and spleen (pooled) as indicated using a cultured ELISPOT assay (n = 4 mice/time point). Data are mean ± s.d. of triplicate cultures using cells. Significantly decreased responses at week 32 compared to the 2-week time point using a one-way ANOVA and Bonferroni correction. **P < 0.01 and ***P < 0.001. (**b**) Schematic representation of the prime–boost vaccination protocol. Mice were immunized three times with the nApa-DDA/MPL vaccine as described in (**a**). Eight months after the last subunit dose, a group of subunit-vaccinated and age-matched naïve mice received a single BCG vaccine-boost (10^6^ CFU) by a homologous i.n. route. Three weeks following a BCG-boost, mice (n = 4/group) were sacrificed and the cellular immune response was investigated. (**c**,**d**) Frequencies of (**c**) nApa- and (**d**) WCL-specific IFN-γ, IL-17 and IL-4-producing cells in the lung and spleen (pooled) using a cultured ELISPOT assay. Data are mean + s.d. of triplicate cultures. Significant using the ANOVA and Bonferroni correction relative to the respective control group. A numbers in the parenthesis indicates a fold change in the total SFU in BCG-boosted group over levels in the respective control group. i.n., intranasal; vac, vaccine; WCL, whole cell lysate of *Mtb*; SFU, spot forming units.

Since BCG can cause severe disseminated disease in immunocompromised individuals, evaluation of a novel subunit-prime followed by selective BCG-boost has been proposed for infants of HIV-infected mothers^30^. We investigated the impact of altering the order of primer and booster vaccine on boosting outcome by administering a single BCG-boost in the subunit Apa-vaccinated mice by a homologous i.n. route during the contraction-phase (week 32) (Fig. 4a,b). Subunit Apa vaccination of mice involved three doses of nApa or rApa (10 μg) in DDA-MPL adjuvant at 2-week intervals by the i.n. route. A single BCG-boost 8 months after the last nApa-subunit dose offered only a modest or no significant boost in nApa- or WCL-specific responses 3 weeks after boosting, relative to levels in non-boosted controls (Fig. 4c,d). In contrast, BCG vaccination of age-matched naïve mice significantly expanded specific cytokine-producing cells during the same period in the lungs and spleen (*P* < 0.05). Similar boosting dynamics were observed when a BCG-boost was administered in rApa-vaccinated mice (Supplementary Fig. S3a–c). These results suggest that subunit Apa-primed protective immunity^13^ likely blocks the growth of BCG in mice and thereby limits the subsequent booster response.

### Homologous route parenteral BCG-prime–Apa-subunit-boost regimen imparts a stronger boost than the mucosal prime-boost approach during the contraction phase

**Figure 5:**
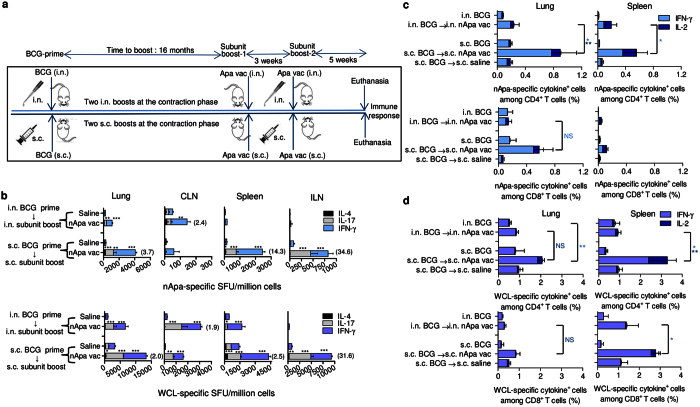
Parenteral BCG-prime and nApa-subunit-boost by a homologous route impart a stronger specific cellular booster effect in the pulmonary and systemic compartments. (**a**) Schematic representation of the prime–boost vaccination regimens employing the homologous parenteral or mucosal routes. Mice were primed once with BCG vaccine by the i.n. or s.c. route. Sixteen months later, mice received two booster doses of nApa (1 μg) in a DDA/MPL adjuvant at a 3-week interval by a homologous route. The BCG-primed mice which were left untreated or boosted (×2) with a saline served as controls. Five weeks after the last subunit-boost, mice (n = 4–5/group) were euthanized and the magnitude and quality of the cellular and humoral immune response was investigated. (**b**) The nApa- and WCL-specific IFN-γ, IL-17 and IL-4 response in the lung, CLN, spleen or ILN (pooled) using a cultured ELISPOT assay. Data are SFU/10^6^ organ cells and error bars represent s.d. of triplicate cultures. Significant using a one-way ANOVA followed by Bonferroni correction comparing cytokine responses of nApa vaccine-boosted groups with those of respective saline-boosted controls. A number in the parenthesis indicates a fold change in the total cytokine SFU in the nApa-boosted group with a higher response over the nApa-boosted group with a lower response (i.e., comparing the nApa vaccine-boosted two regimens employing different routes). (**c,d**) The frequency (%) of nApa-specific (**c**) and WCL-specific (**d**) IFN-γ or IL-2-producing cells among CD4^+^ and CD8^+^ T lymphocytes of the lung and spleen using a flow cytometry. Data are means + s.e.m. (n = 4–5 individually analyzed mice/group). *P < 0.05; **P < 0.01 and ***P < 0.001 using the ANOVA and Bonferroni correction (in **b–d**). s.c., subcutaneous; i.n., intranasal; vac, vaccine; WCL, whole cell lysate of *Mtb*; SFU, spot forming units.

BCG-prime–Apa-subunit-boost regimen employing a homologous route elicited a stronger booster responses than the regimen employing heterologous routes (Figs 2 and 3). We compared the magnitude and distribution of booster responses elicited by homologous s.c.–s.c. route regimen with those induced by i.n.–i.n. regimen. We administered two Apa-subunit-boosters in s.c. or i.n. BCG-primed mice, each using lower (1 μg) protein dose, 3 weeks apart at 16 months post-BCG-priming, by the corresponding homologous route (Fig. 5a), whereby the booster vaccine drained to the same LNs as the primer vaccine. Five weeks after the second booster, cytokine and antibody responses were investigated.

We found a significant increase in nApa- or WCL-specific response (IFN-γ, IL-17 and IL-4 SFU) in the lung, spleen and draining LNs of mice receiving nApa compared to those receiving saline-boosters (Fig. 5b). Interestingly, specific total SFU were 2.0–14.3-fold greater in the lungs and spleen of mice receiving s.c.–s.c. regimen than those receiving i.n.–i.n. regimen (Fig. 5b). These results suggest that although nApa-specific cells contract more in s.c. BCG-primed mice during waning (Fig. 1a), they undergo greater-fold expansion in pulmonary and systemic compartments following a homologous route nApa-boost. Since mice received s.c. BCG on their hind legs, responses in the inguinal lymph nodes (ILN), which drains flanks, were boosted exclusively in s.c.–s.c. regimen. Conversely, specific responses in the CLN, which drains the upper-respiratory tract, were boosted substantially in the i.n.–i.n. regimen (Fig. 5b). These results confirm the route-specific compartmentalization of BCG-primed responses and its effect on the distribution and geography of booster responses.

Simultaneously, we quantified frequencies of IFN-γ or IL-2-producing T cells in the lung and spleen and found about 2.0–4.7-fold greater frequencies of nApa- or WCL-specific cytokine-producing CD4^+^ and CD8^+^ cells in mice receiving the prime–boost regimen by a homologous s.c. rather than i.n. route (Fig. 5c,d), with the exception of nApa-specific splenic CD8^+^ T-cell response. The splenic CD4^+^ T-cell responses of nApa-boosted mice consisted of significant proportions of nApa- and mycobacteria-specific IL-2-producing cells. Considering the almost complete disappearance of nApa-specific IL-2^+^ cells during the contraction-phase of BCG-response, this amplified frequency of these T cells following nApa-boosts suggest modulation of the antigen-specific T-cell function following a heterologous subunit-boost.

To address why nApa- but not rApa-boost effectively increased the IL-2 response in BCG-primed mice (Supplementary Fig. S2c,d), we probed the epitope specificity of IL-2 response by screening 32 overlapping synthetic Apa peptides in the ELISPOT assay after a s.c. nApa-boost. No single synthetic peptide induced a strong IL-2 response (Supplementary Fig. S4a), despite heightened C-terminal peptides-specific IFN-γ, IL-17 and IL-4 responses. Corroborating IL-2 ELISPOT results, we found no significant increase in specific IL-2^+^CD4^+^ T-cell frequency following rApa-boost, despite its comparable ability to boost specific IFN-γ, IL-17 and IL-4 SFU and IFN-γ^+^CD4^+^ T cells in the lung (Supplementary Fig. S4b,c), which highlights the differential influence of the Apa-form on the booster IL-2 response. We also investigated cytokine production by T-cell hybridomas generated against nApa^17^. Upon co-culture with nApa-pulsed dendritic cells a significant amount of IL-2 was produced by hybridoma clones specifically recognizing only nApa or its N-terminal glycopeptide compared to clones recognizing both nApa and rApa (Supplementary Fig. S5a,b). Interestingly, none of these nApa-specific clones produced significant IFN-γ (data not shown), an observation similar to glycopeptide-specific memory T-cell response reported after protective streptococcal-glycoconjugate vaccination^31^. These results together with the preferential specific IL-2 response in nApa-boosted BCG mice collectively indicate that nApa mannopeptide-specific T cells are capable of expressing an IL-2-producing phenotype.

**Figure 6:**
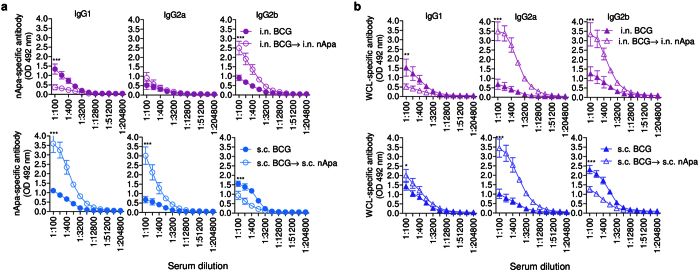
Homologous route parenteral BCG-prime–subunit-nApa-boost regimen induces a distinct magnitude and quality of specific serum antibody response compared to the mucosal regimen. (**a,b**) Serum IgG-subclass antibody responses of the i.n.–i.n. and s.c.–s.c. regimen vaccinated mice described in Fig. 5a using ELISA. The nApa- (**a**) and WCL-specific (**b**) serum IgG1, IgG2a and IgG2b levels are plotted in comparison with the corresponding non-boosted controls. Error bars at 1:100, 1:200 and 1:400 serum dilutions are means ± s.d (n = 4 mice/group). *P < 0.05; **P < 0.01 and ***P < 0.001 using the ANOVA and Bonferroni correction. s.c., subcutaneous; i.n., intranasal; vac, vaccine; WCL, whole cell lysate of *Mtb.*

Emerging evidence suggests a role for vaccination-induced antibodies in protection against *Mtb*^32^. Thus, we investigated the serum antibody response following a subunit-boost using ELISA. In BCG-primed mice, mycobacteria-specific antibody levels peak at 8 months and wane significantly by 16 months^8^. The timing and route of a subunit nApa-boost in BCG-primed mice markedly influenced the magnitude and quality of antibody response (Supplementary Fig. S6a,b). A striking difference in the quality of specific serum IgG subclass-antibody response was also observed between s.c.–s.c. and i.n.–i.n. regimens (Fig. 6a,b), suggesting a differential influence of parenteral versus mucosal prime–boost regimen on the IgG-subclass switching during the booster antibody response.

Overall, these results demonstrate that the low dose nApa-boosters by homologous parenteral route via skin are sufficient to expand specific cell-mediated and antibody responses compared to homologous mucosal airway boosts in BCG-primed mice.

### Parenteral nApa-boost by a homologous route during the contraction-phase of BCG-immunity maintains IL-2^+^CD4^+^ T cells and improves waning protection against *Mtb* infection

**Figure 7:**
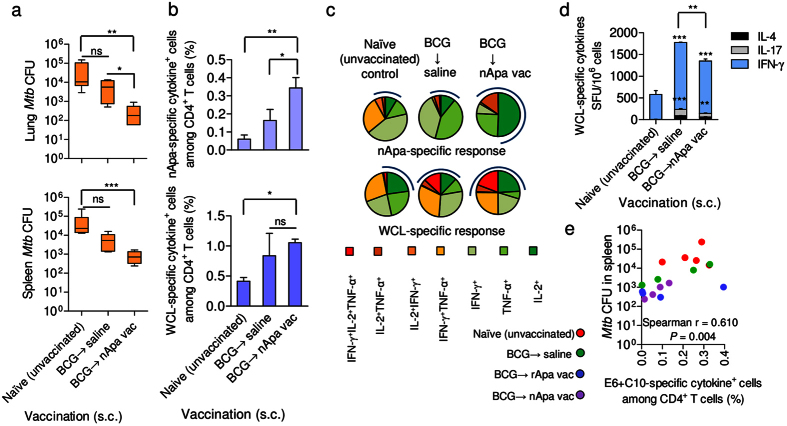
Subunit nApa-boosters by a homologous s.c. route during the contraction-phase improve waning BCG-induced protection against *Mtb* challenge in the elderly mice. (**a**) Protective efficacy of the nApa- or saline-boosted BCG mice. Mice were prime–boost vaccinated by the s.c. route as depicted in Fig. 5a. The *Mtb* bacillary load in the lung and spleen 6 weeks post-challenge is plotted as box plots (n = 5–6 mice/group). CFU were determined using whole lungs and spleens. Whiskers indicate maximum and minimum CFU levels. Significant by the Kruskal-Wallis and Dunn’s test compared to the age-matched unvaccinated naïve controls; ns: non-significant. **P < 0.01 and ***P < 0.001. The remaining splenocyte suspensions after plating five different dilutions on Middlebrook-7H10 agar plates for *Mtb* CFU enumeration were used for immune response studies. (**b,c**) Magnitude (**b**) and quality (**c**) of nApa- and WCL-specific total cytokine (IFN-γ, IL-2 and/or TNF-α)-producing cells among splenic CD4^+^ T lymphocytes post-challenge using a flow cytometry. (**b**) Histograms represent mean + s.e.m. responses (n = 4 randomly selected and individually analyzed mice/group in **a**). Significant using ANOVA followed by the Bonferroni correction. (**c**) Pie-charts show proportions of seven subpopulations of cytokine-producing CD4^+^ T cells in any combination constituting total specific CD4^+^ T-cell response based on the cytokine co-expression profiles. Pie charts present mean frequencies of seven cytokine subsets (n = 4 mice/group in **b**) and arcs denote the IL-2-producing cells in any combinations. (**d**) Frequencies of WCL-specific IFN-γ, IL-17 and IL-4 SFU/million splenocytes using a cultured ELISPOT assay. Data are mean ± s.d. of triplicate cultures. Significant using ANOVA followed by the Bonferroni correction after comparing responses of two boosted groups. (**e**) Frequency of ESAT-6 + CFP-10-specific total cytokine-producing cells among CD4^+^ T lymphocytes as a surrogate for bacterial burden. Each symbol represents response of one mouse (4–5 mice/group in **a**). Significant using Spearman rank correlation test. s.c., subcutaneous; vac, vaccine; WCL, whole cell lysate of *Mtb*; SFU, spot forming units.

When we investigated the ability of BCG-vaccinated mice to protect against *Mtb* infection at the peak versus contraction-phase, a stronger and comparable protection was found at 8 months (1.93–2.45-log CFU-reduction in lung and spleen), regardless of i.n. or s.c. priming^8^, which left a little scope to improve upon BCG-efficacy using a subunit-boost in this model. Conversely, BCG-induced protection was lost completely by 18 months, which indicated the necessity to boost waning immunity^8^,^33^. Since the homologous s.c.–s.c. regimen afforded a stronger boost in waning BCG-elicited responses, we investigated the ability of a s.c. Apa-booster (2×) regimen (Fig. 5a) to impart protection against virulent *Mtb* challenge. Boosting BCG-responses after 16 months with nApa significantly improved protection (Fig. 7a), with 1.2–1.5-log CFU-reduction in the spleen (*P* = 0.0019) and lung (*P* = 0.0084) 6 weeks post-challenge in nApa-boosted compared to age-matched naïve controls. A 0.6–1.2-log CFU-reduction in nApa-boosted relative to saline-boosted BCG mice was also observed (*P* < 0.05 in the lung). These results demonstrate the potential of a subunit nApa-boost by homologous parenteral route to improve waning BCG-induced protection in elderly mice.

Simultaneously, when we investigated possible differences in the magnitude and quality of T-cell responses among test and control groups using splenocytes of individual mice, we found significantly greater frequencies of nApa-specific total cytokine-producing (IFN-γ, IL-2 and/or TNF-α) CD4^+^ T cells in nApa-boosted mice (*P* = 0.0079) compared to saline-boosted and age-matched naïve controls (Fig. 7b). Although frequencies of mycobacteria (WCL)-specific CD4^+^ cells were also higher in nApa-boosted mice, the response was significant only relative to age-matched naïve controls (*P* = 0.074). Furthermore, while WCL- or nApa-specific CD4^+^ T-cell responses of nApa-boosted mice were characterized by greater proportions (40–60%) of IL-2-producing cells (IL-2^+^, IL-2^+^TNF^+^ and/or IL-2^+^TNF-α^+^IFN-γ^+^) after *Mtb* infection (Fig. 7c), those of age-matched naïve and saline-boosted control mice consisted of higher proportions (60–80%) of IFN-γ and/or TNF-α-producing cells only. The mycobacteria-specific cultured ELISPOT responses of all three groups were dominated by IFN-γ (Fig. 7d), but, significantly less *Mtb*-specific cytokine SFU were found in nApa-boosted compared to saline-boosted group. Importantly, the frequency of ESAT-6+CFP-10-specific cytokine-producing CD4^+^ T cells investigated as a marker of *Mtb*-load and pathology correlated directly with *Mtb* burden (Spearman r = 0.610, *P* = 0.004), with significantly higher ESAT-6+CFP-10-specific cytokine^+^CD4^+^ T cells in controls than nApa-boosted mice (Fig. 7e).

Contrary to nApa-boosted mice, rApa-boosted group had low frequencies of nApa- or WCL-specific IL-2-producing CD4^+^ T cells before challenge and no significant expansion in IL-2^+^CD4^+^ T cells occurred post-challenge (Supplementary Fig. S7a). The T-cell responses of rApa-boosted mice presented with a greater frequency of nApa-specific TNF-α-producing and WCL-specific IFN-γ^+^TNF-α^+^ CD4^+^ T cells (Supplementary Fig. S7a, pies). These mice also maintained relatively higher mycobacteria-specific IFN-γ, IL-17 and IL-4 SFU post-challenge (Supplementary Fig. S7b). Despite these notable differences in the magnitude and quality of pre- and post-challenge responses, nApa- and rApa-boosted mice presented with comparable *Mtb* burdens and both Apa-vaccine forms imparted a similar degree of protection in the 6-week experiment (Fig. 7a and Supplementary Fig. S7c). Considering the important role of specific IL-2^+^CD4^+^ T_CM_ cells in the control of chronic *Mtb* infection^25^, these results suggest a need to further evaluate the sustainability of nApa and rApa-booster vaccination-induced protection in the long-term protection experiments.

It should be noted that comparable to nApa subunit-vaccination, significant proportions of rApa vaccination-induced CD4^+^ T-cell response consisted of nApa- and mycobacteria-specific IL-2-producing cells in the spleen and/or ILN (particularly CD4^+^IL-2^+^TNF-α^+^ T cells) (Supplementary Fig. S8). In fact, a significantly greater frequency of nApa-specific IL-2^+^TNF-α^+^CD4^+^ T cells expanded in the spleen of rApa- compared to nApa-vaccinated mice post-challenge. These results suggest that the weak IL-2^+^CD4^+^ T-cell responses in BCG-primed–rApa-boosted mice were not due to the inherent inability of rApa vaccine to induce and expand specific IL-2 responses but is likely due to prior BCG-primed milieu where responses are primed mainly against glycosylated nApa.

## Discussion

A key question in preclinical and clinical trials evaluating BCG-booster vaccines is when to administer a booster. In children, one option is to boost shortly after BCG vaccination, coinciding with the existing schedule of Expanded Programme on Immunization^7^,^34^. However, co-administration of multiple vaccines during infancy could lead to immunological interference, as reported for MVA85A^35^. Another potentially useful time to boost BCG is in adolescence, which is many years after BCG vaccination when BCG-immunity is thought to wane and before rise in the incidence of TB disease that occurs in adolescents and young adults. Since an effective booster vaccine is expected to improve waning BCG-immunity to afford long-lasting protection and because boosting T-cell responses during the expansion-phase may lead to activation-induced cell death or drive terminally-differentiated, exhausted and short-lived effector T cells^7^,^11^,^12^,^36^, in this study we investigated the potential of Apa vaccine to boost BCG-induced responses after expansion-phase, i.e., at the peak of response or during waning of primary immunity.

Our data indicate that boosting parenteral or mucosal BCG-primed response with a subunit vaccine is optimal after a prolonged interval, especially when the primary effector response subsides. Indeed, longer time-intervals between the prime and boost with heterologous vaccines have been shown to optimize both antibody and T-cell responses in animal models and humans with a variety of vaccine platforms^37^,^38^. Our results demonstrate that boosting BCG-responses with a subunit vaccine during the contraction-phase imparts a higher-fold boost in specific responses compared to boosting at the peak of the effector response. Although specific responses were subsiding at the time of boost during the contraction-phase (i.e., at 16 months) in our study, they did not wane completely and efficient T_EM_ responses were generated^8^. Conversely, at the peak of BCG-response, robust frequencies of antigen-specific IFN-γ^+^Th1 cells persisted. It is known that IFN-γ can regulate IL-17 response^39^, and such highly activated IFN-γ^+^Th1 effector cells are more prone to proliferation- and activation-induced cell-death than Th2 and Th17 cells following re-stimulation^36^,^40^,^41^. Speculatively, increased apoptosis of these specific T cells following a subunit-boost might be one of the reasons for the suboptimal booster effect observed at the peak of response.

Reinforcing our findings, the timing of subunit-HBHA-boost after BCG-priming has been previously shown to influence the boosting outcome^42^. A modest enhancement over prior BCG-immunity was observed when the short prime to boost interval (i.e., 3 weeks or 4 months) was used in mice, in contrast, prolonging the interval to 8 months led to a stronger booster response and improved protection in-terms of reduction in *Mtb* burden^42^. However, systematic kinetics of HBHA-specific T-cell response in BCG-primed mice was not investigated^42^, and since BCG-induced protection itself peaks around 8 months post-priming^8^,^33^, the impact of boosting during the expansion versus contraction-phase could not be clearly comprehended. Recently, boosting BCG-primed responses during the effector-phase has been suggested as one of the reasons for failed protection using Ag85A vaccine^43^, and administration of two Ag85A-boosters, one at the peak and other during waning, has been shown to improve protection^33^. In contrast, the boosting potential of MVA85A was not found to be influenced by the timing of the boost after BCG-priming in children^34^, however, the authors of this study have cited several limitations in their study design. Interestingly, *Mtb*72F/AS02A-boost in cynomolgus monkeys has been shown to afford superior protection when it is administered at 4 months compared to 8 months after BCG-priming^44^. This observation is at odds with our proposition to use prolonged boosting intervals. It is likely that the timing of the boost in BCG-primed hosts may not be critical for some vaccines. Such candidates may include latency-associated antigens, polypeptides with reduced expression following early bacillary-growth (example Ag85B) or antigens which are absent in BCG (example ESAT-6)^10^. The differences in antigen-specific response kinetics, vaccination platform and/or host system used might be responsible for these different outcomes. Nonetheless, these findings together with our results highlight the importance of the optimization of immunization schedule, especially the time-interval between prime and boost.

It is currently not known exactly which T-cell responses BCG-booster vaccines should induce for improved protection. While heightened specific IFN-γ response in heterologous prime‒boost regimens has been shown to correlate with enhanced protection^20^,^42^, increasing evidence questions the value of IFN-γ as a sole correlate of protection^45^,^46^. In our study, lengthening the time-interval between BCG-prime and Apa-boost was associated with induction of balanced proportions of specific IFN-γ and IL-17-producing cells and heightened frequency of IL-2-producing cells. Previously, the propensity of T-cells to induce IL-17 response has been shown to increase with increasing age^47^, and induction of balanced type-1 and type-17 responses has been shown to correlate with superior protective efficacy of rBCG vaccine^48^. Mucosal Apa-boost (10 μg) during the contraction-phase also enhanced the specific IL-4 response in the lung, regardless of the form of Apa-booster used. However, the frequency of nApa-specific IL-4-producing cells was about 2-fold less than the specific IL-17 or IFN-γ-producing cells. Further studies are required to understand the significance of Th1 and Th17 balance, Th1 and Th2 balance or for that matter balance between the pro-inflammatory and anti-inflammatory cytokine responses induced by BCG-prime–subunit-boost vaccination regimens in protection. In view of the near absence of specific IL-2-producing cells during the contraction-phase, a heightened IL-2 response resulting from subunit-boosting suggests the capability of a nApa-boost to modulate functions of preexisting specific T cells and/or imprint an altogether new T-cell response. Since the nApa but not rApa-booster increased IL-2 response in BCG-primed mice during waning, it is tempting to speculate that nApa carbohydrate/glycopeptide-specific long-lived memory T-cells are generated in BCG mice and are effectively boosted following nApa-boost. Alternatively, such increased nApa-booster elicited IL-2 response may represent protective immune-regulatory mechanisms to counterbalance persistent BCG-elicited immune activation^49^. While the memory phenotype after the nApa-boost was not evaluated in our study, the subunit vaccine-induced IL-2^+^TNF-α^+^ CD4^+^ T cells have been recently shown to be associated with CD62L^+^T_CM_ phenotype and greater expansion potential following *Mtb* challenge and protection^25^. Since BCG vaccination predominantly induces IFN-γ/Th1 response and further boosting of Th1 response using MVA85A vaccine has recently failed to improve protection^43^, we propose that novel BCG-booster vaccines should re-educate the immune system rather than boosting a prior Th1 immunity.

Specific T-cell responses in pulmonary as well as systemic compartments are likely required for superior protection against pulmonary TB and prevention of dissemination. Another factor that strongly influenced the magnitude and distribution of the booster response in our study was the route of prime‒boost vaccinations. The homologous s.c. route regimen, where BCG-primer and nApa-booster drained to the same LNs, afforded stronger pulmonary and systemic boost in specific CD4^+^ T cells compared to the homologous i.n. regimen, and improved waning BCG-immunity in elderly mice. Such a strong induction of pulmonary T-cell responses by a non-mucosally administered regimen in our study is rather striking. Previous reports of strong pulmonary responses involved the use of the respiratory route and live multiplying viral-vectored vaccines^20^,^21^,^22^. Our results could be partly explained on the basis of the fact that BCG-priming via skin is fully capable of inducing strong systemic T-cell response with a lung-homing phenotype and sound T-cell immunity in the lung interstitial tissue (despite modest response in the airway lumen)^50^. Furthermore, a protein vaccination by mucosal route may suffer from an increased proteolytic degradation, decreased immunogenicity and need for a greater vaccine dose^51^. The lack of a protection experiment comparing s.c. versus i.n. prime‒boost regimen can be cited as a limitation of our study. However, about 10-fold higher Apa dose is indeed required via i.n. route to impart comparable protection to s.c. subunit vaccination using DDA/MPL adjuvant (unpublished data), and the side effects of robust inflammation induced by strong adjuvanted vaccine in the respiratory tract may preclude the use of prime‒boost regimens via the respiratory tract^51^,^52^.

Recently, a role for Apa glycosylation in *Mtb* virulence has been suggested^16^,^17^,^53^ and overexpression of *Mtb* nApa in BCG was shown to abrogate protection conferred by BCG^53^. It remains to be seen whether nApa-boost during the expansion-phase, when higher BCG-load persists in the draining LNs and spleen^8^,^27^ abolishes BCG-induced protection. However, no loss or improvement in BCG-induced protection was reported following microparticle-encapsulated *apa* genetic-boost during the expansion phase in a short-term protection experiment, and in fact, *apa*-boost modestly improved protection in the lung against chronic *Mtb* infection and prevented pathology^54^. In our study, subunit nApa-boost generated a strong booster effect despite targeting the same draining LNs where BCG persists^27^. Our preliminary data suggest that nApa but not rApa or saline-boost during the contraction-phase of BCG-immunity resist *Mtb* infection-driven increase in terminally-differentiated KLRG-1^+^CD4^+^ T cells post-challenge (data not shown). Despite notable differences in IL-2 and IL-17 responses induced by nApa and rApa-booster regimens post-challenge, a similar degree of protection was observed with two booster vaccines in a 6-week infection experiment. Maintenance of specific IL-2^+^CD4^+^ or IL-17-producing T cells post-challenge in BCG-primed–subunit-boosted mice have been shown to correlate with improved protection^25^,^55^. Since excess IL-17 induction in response to repeated mycobacterial vaccination can result in severe lung pathology^56^, further long-term protection studies comparing two booster vaccines against chronic *Mtb* infection with histopathological investigations are needed. However, several factors complicate such prolonged studies during waning in this model, including increased mortality due to chronic BCG-burden and advancing age, poorly understood age-related immunological changes and the known capability of older mice to efficiently control early *Mtb* infection^8^. Therefore, our results together with recent observations^8^,^27^ highlight the conundrum of optimization of subunit-booster regimens in BCG-primed mice, where BCG chronically persists and drives persistent immune activation. One way to circumvent this issue could be to evaluate simultaneous BCG and subunit vaccination^57^ or BCG supplementation^58^. Such vaccination approaches could aim to imprint distinct immunity by improving T_CM_ and other protective responses insufficiently produced by BCG^3^. Alternatively, we propose that preclinical prime–boost regimens can be best investigated in higher animal models (example, nonhuman primate) considering the limitations of mouse model.

In humans, the persistence of BCG is not known with certainty. Reports of BCG-lymphadenitis or dissemination years after BCG vaccination are used to support a relatively long life-span of this vaccine^59^,^60^. Since any novel BCG-booster vaccine in humans will need to recognize the high prevalence of latent *Mtb* infections and repeated low dose mycobacterial exposures in highly-endemic regions^3^, our study points out the challenges of optimization of BCG-booster regimens in this population in the context of immune-activation generated by BCG and subsequent exacerbation by *Mtb* infections. Overall, our results have important implications for the development of effective prime–boost vaccination strategies against TB.

## Methods

### Mice

Specific-pathogen-free 6–8-week-old female BALB/c mice (Charles River, Wilmington, MA) were used in this study. All procedures were approved by the Institutional Animal Care and Use Committee of Centers for Disease Control and Prevention, Atlanta and performed as per the guidelines of U.S. Public Health Service Policy on the Humane Care and Use of Animals.

### Antigens

Native Apa was purified from the *Mtb* H37Rv culture filtrate by a traditional and reversed-phase chromatography, while the recombinant Apa was expressed in *E. coli* BL21 (DE3) and purified using the combinations of Nickel and DEAE-Sepharose column chromatography techniques as described previously^14^,^17^. The rApa clone pMRLB.17 and WCL of *Mtb* H37Rv were obtained through the NIH Biodefense and Emerging Infection Research Resources Repository.

### Vaccinations and infections of mice

BCG-vaccinated mice received 10^6^ CFU Copenhagen by s.c. or i.n. route^8^. For subunit-vaccinations, mice received 1–3 doses of nApa or rApa (1 or 10 μg) in a DDA-MPL adjuvant (250 μg DDA and 25 μg MPL-A/dose; Sigma-Aldrich, St. Louis, MO)^13^,^17^ as described in the schematics of regimens. *Mtb* infections were performed by the airway i.n. route using 5 × 10^4^ CFU Erdman^17^. At 48 h following challenge, about 250 mean CFU could be cultured from the lungs. The *Mtb* bacillary burden was determined by plating organ homogenates onto the Middlebrook-7H10 agar supplemented with the oleic acid–albumin–dextrose–catalase (Becton Dickinson, San Diego, CA) and 2-thiophene-carboxylic acid hydrazide (2 μg/ml). The CFU were enumerated after 4 weeks of incubation at 37 °C. Detailed procedures of mice vaccinations, euthanasia and tissue isolation are available in the Supplementary Information.

### Flow cytometry

Cell surface markers and the intracellular cytokine expression of organ immune cells were assessed using a polychromatic flow cytometry^8^,^17^, following an *in vitro* stimulation with the *Mtb* antigen (10 μg/ml) at 37 °C for a total 12 h in the RPMI-1640 medium. A detailed procedure is available in the Supplementary Information.

### ELISPOT assay

IFN-γ, IL-4 and IL-17A ELISPOT assays were performed using a commercially available mouse ELISPOT reagent sets (BD-Biosciences) and by using *Mtb* antigens (10 μg/ml) for *in vitro* stimulations of organ cells at 37 °C for 40 h^8^,^17^. A detailed procedure is available in the Supplementary Information.

### Antibody ELISA

Antibody levels in the mouse serum were estimated using the nApa (2 μg/ml) or WCL (5 μg/ml) for coating and the anti-mouse IgG1, IgG2a (BD-Pharmingen) and IgG2b (Santa Cruz Biotechnology, Dallas, TX) HRP-conjugated antibodies for detection.

### T-cell hybridomas and cytokine ELISA

T-cell hybridomas specific for nApa were generated using a draining LN cells of the nApa-vaccinated mice and a T-cell fusion partner BWα^−^β^−^17. Hybridoma clones were screened using a bone-marrow-derived dendritic cells or the B-cell lymphoma line A20 pulsed with 0.5 μg/well nApa. The estimation of cytokines secreted by the responding T-cell hybridomas was performed by assaying a 24-h culture supernatants using paired rat mAbs specific for the mouse IL-2 or IFN-γ (BD-Biosciences/Pharmingen) in a capture ELISA. Further details are available in the Supplementary Information.

### Statistical analyses

Differences between groups were assessed by a 1-way ANOVA with Bonferroni correction or by a nonparametric Kruskal-Wallis test followed by the Dunn’s post-test (GraphPad Prism, version-5). Unless indicated, all immune response data are presented after subtracting no antigen control values. *P* values < 0.05 were considered statistically significant.

## Additional Information

**How to cite this article**: Nandakumar, S. *et al.* Boosting BCG-primed responses with a subunit Apa vaccine during the waning phase improves immunity and imparts protection against *Mycobacterium tuberculosis*. *Sci. Rep.* **6**, 25837; doi: 10.1038/srep25837 (2016).

## Supplementary Material

Supplementary Information
