# Supplementary material for: Boosting BCG-primed responses with a subunit Apa vaccine during the waning phase improves immunity and imparts protection against Mycobacterium tuberculosis
Source: Sci Rep. 2016 May 13;6:25837. doi: 10.1038/srep25837 (PMC4865829; doi:10.1038/srep25837)
Supplement: Supplementary Information [file srep25837-s1.pdf]

Supplementary Information

Boosting BCG-primed responses with a subunit Apa vaccine during the waning phase improves immunity and imparts protection against *Mycobacterium tuberculosis*

Subhadra Nandakumar[1], Sunil Kannanganat[2], Karen M. Dobos[3], Megan Lucas[3], John S. Spencer[3], Rama Rao Amara[2], Bonnie B. Plikaytis[1], James E. Posey[1], and Suraj B. Sable[1*]

[1]Division of Tuberculosis Elimination, National Center for HIV/ AIDS, Viral Hepatitis, STD, and TB Prevention, Centers for Disease Control and Prevention, Atlanta, GA 30333, USA; [2]Department of Microbiology and Immunology, Emory Vaccine Center and Yerkes National Primate Research Center, Emory University, Atlanta, GA 30329, USA; [3]Department of Microbiology, Immunology and Pathology, Collage of Veterinary Medicine and Biomedical Sciences, Colorado State University, Fort Collins, CO 80523, USA.

* ***Correspondence.***

Suraj B. Sable, DVM, PhD.

Centers for Disease Control and Prevention

Email: SSable@cdc.gov

Phone: 404-639-1478

Fax: 404-639-1287

Supplementary figures

Figure S1. Longitudinal changes in the IFN-γ, IL-2 or TNF-α expression by *M. tuberculosis* Apa-specific T cells after mucosal or parenteral BCG vaccination.

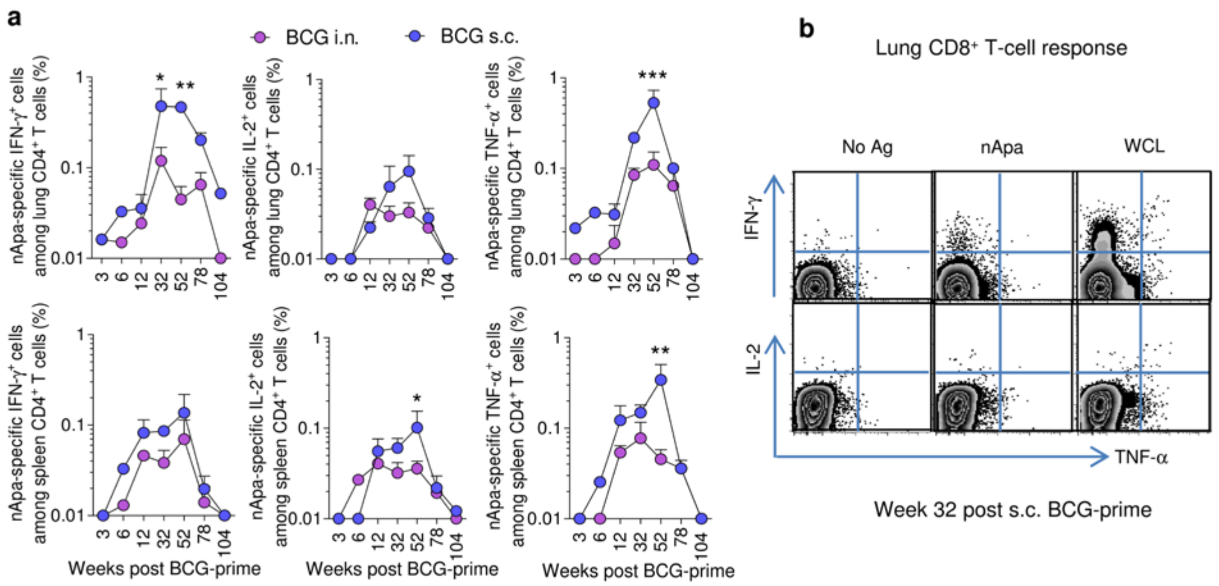

Mice were vaccinated once with BCG by the i.n. or s.c. route as described in **Figure 1**. At different time points after vaccination, as indicated, mice were euthanized (n = 4 mice/time point/group) and their lungs and spleen were harvested. Cytokine expression by T cells was measured by an intracellular cytokine staining and a polychromatic flow cytometry following the stimulation of spleen or lung cells (pools) with a medium alone (no antigen), nApa or WCL. (**a**) Kinetic changes in the frequencies of nApa-specific $CD4^+$ T cells expressing IFN-γ, IL-2 or TNF-α. Data are percentages (%) of nApa-specific cytokine-producing cells among $CD4^+$ T cells plotted for an individual cytokine after subtracting no antigen stimulation control values. Data at week 12, 32, 52 and 78 are means ± s.e.m. of 3–4 independent mouse experiments, while data (means) at week 3, 6 and 104 are from one experiment evaluated in duplicate. *$P < 0.05$, **$P < 0.01$ and ***$P < 0.001$ comparing responses of i.n. and s.c. BCG using one-way ANOVA followed by Bonferroni's post-test. (**b**) Representative flow-cytometric

plots from one experiment showing nApa or WCL-specific IFN-γ, IL-2 and/or TNF-α expression by CD8^{+} T cells in the lung at the peak of response (week 32) after s.c. BCG vaccination. s.c., subcutaneous; i.n., intranasal; Ag, antigen; WCL, whole cell lysate of *Mtb*.

Figure S2. Timing, route and form of a subunit-Apa-boost in the BCG-primed mice impact the cellular booster response

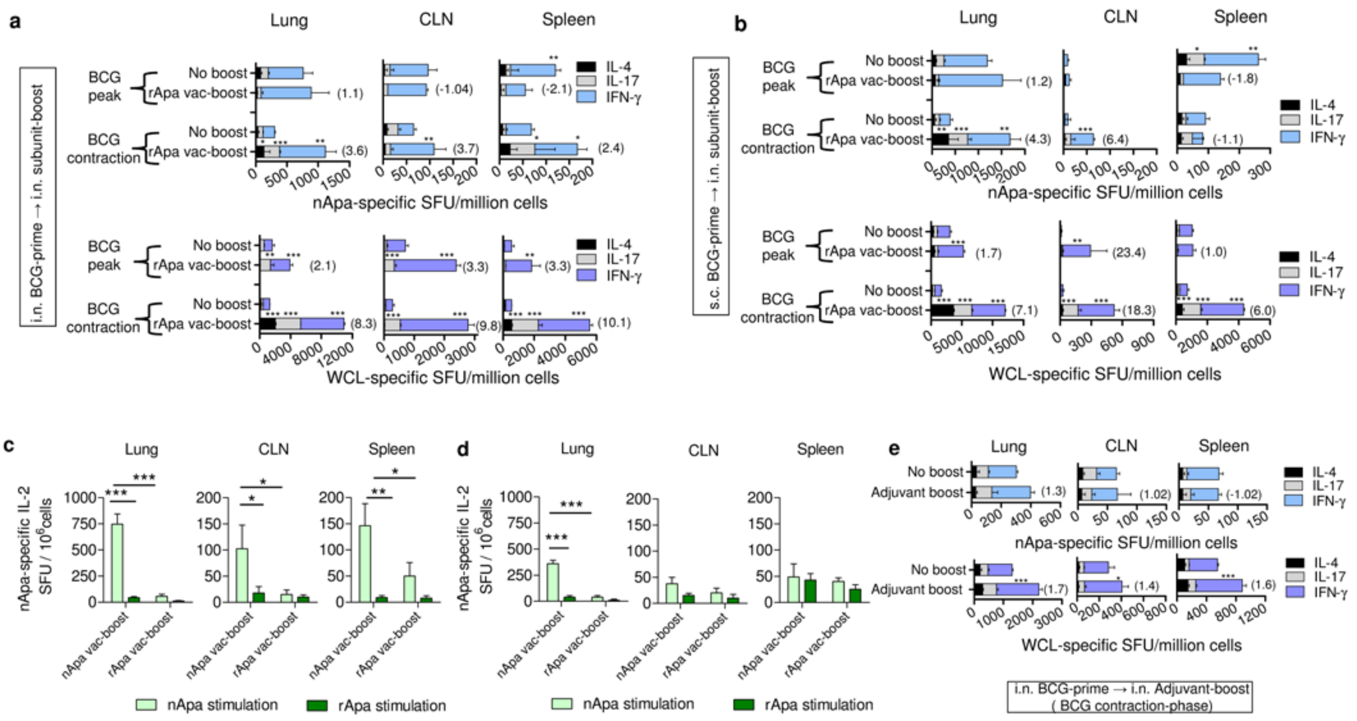

Mice were primed with BCG vaccine by the i.n. or s.c. route and boosted with a subunit nApa or rApa (10µg) vaccine using a DDA/MPL adjuvant by a homologous or heterologous route at the peak (8 months) or contraction-phase (16 months) of BCG-elicited response. In one experiment, i.n. BCG-primed mice were boosted with a DDA/MPL adjuvant alone by a homologous i.n. route during the contraction-phase. Three weeks after a subunit vaccine- or adjuvant-boost, frequencies of antigen-specific cytokine-producing cells in the lung, CLN or spleen (pooled) were determined using a cultured ELISPOT assay. (**a**–**b**) Influence of the timing and route of a subunit-rApa-boost in the BCG-primed

mice on the cellular booster response. The nApa- (upper panels) and WCL-specific (lower panel) IFN-γ, IL-17 and IL-4 SFU per million organ cells following a single i.n. subunit-rApa-boost in (**a**) i.n. (homologous route) or (**b**) s.c. (heterologous route) BCG-primed mice are plotted. Number in the parenthesis indicates a fold increase in total IFN-γ, IL-17 and IL-4 SFU in rApa-boosted mice over corresponding non-boosted BCG-primed controls. (**c–d**) The form of Apa vaccine (native versus recombinant) influences IL-2 booster response. The IL-2 SFU per million organ cells following nApa or rApa subunit-boost at the contraction-phase of BCG-response using (**c**) a homologous (i.n.–i.n.) or (**d**) heterologous (s.c.–i.n.) route prime–boost regimen. (**e**) Boosting BCG-primed responses with an adjuvant alone does not afford a stronger antigen-specific boost. Error bars in (**a–e**) represent s.d. of cytokine response using pooled organ cells in triplicate cultures (n= 4mice/group/time point). Significant using ANOVA followed by Bonferroni's post-test. **P* < 0.05, ***P* < 0.01 and ****P* < 0.001. s.c., subcutaneous; i.n., intranasal; vac, vaccine; WCL, whole cell lysate of *Mtb*; SFU, spot forming units.

Figure S3. Boosting a subunit rApa vaccine-primed responses with BCG does not afford a stronger boost.

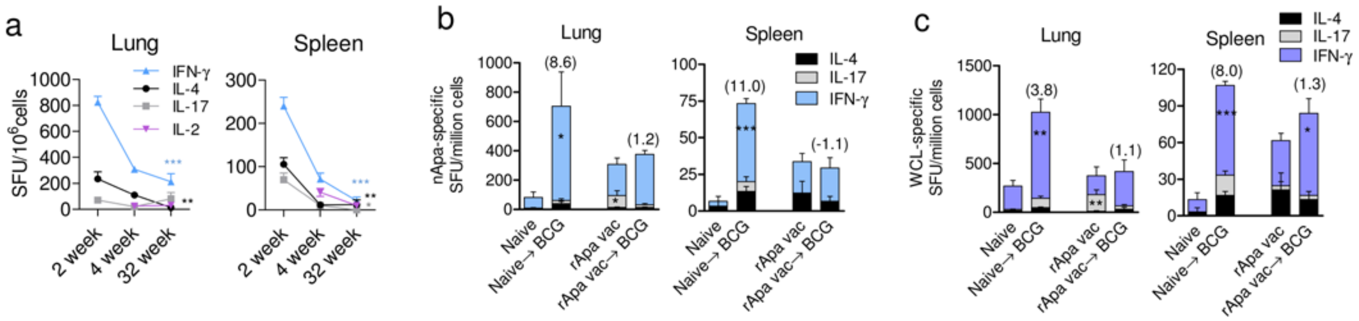

(**a**) The magnitude and kinetics of cytokine-producing cells in rApa vaccinated mice. Six-eight-week old mice were subunit immunized three times with rApa (10µg/dose) in a DDA-MPL adjuvant at 2-week intervals by the i.n. route, and IFN-γ, IL-2, IL-4 and IL-17 responses were investigated in the lung and spleen as indicated using a cultured ELISPOT assay. Data are mean ± s.d. of triplicate cultures using

pooled cells (n = 4 mice/time point). The IL-2 response was not investigated at the 2-week time point. Significant waning of cytokine response at the week 32 compared to the 2-week time point using a one-way ANOVA and Bonferroni's test. (**b–c**) Boosting rApa vaccine-primed responses with BCG. Mice were immunized three times with rApa in a DDA-MPL adjuvant as described in (**a**). Eight months after the last subunit dose, a group of subunit-vaccinated or age-matched naïve mice received a single BCG vaccine-boost (10^6 CFU) by a homologous i.n. route. Three weeks following a boost, mice (n = 4 mice/group) were euthanized and frequencies of (**c**) nApa- and (**d**) WCL-specific IFN-γ, IL-17 and IL-4-producing cells in the lung and spleen (pooled) were determined using a cultured ELISPOT assay. Data are mean + s.d. of triplicate cultures. Number in the parenthesis indicates a fold change in the total SFU in BCG-boosted group over levels in the respective control group. Significant using ANOVA and Bonferroni's test compared to the respective control group. *P < 0.05, **P < 0.01 and ***P < 0.001. i.n., intranasal; vac, vaccine; WCL, whole cell lysate of *Mtb*; SFU, spot forming units.

Figure S4. Synthetic peptide screening in the BCG-primed–Apa-boosted mice reveals the breadth of specific booster response and confirms a differential influence of Apa-form on IL-2 response.

a

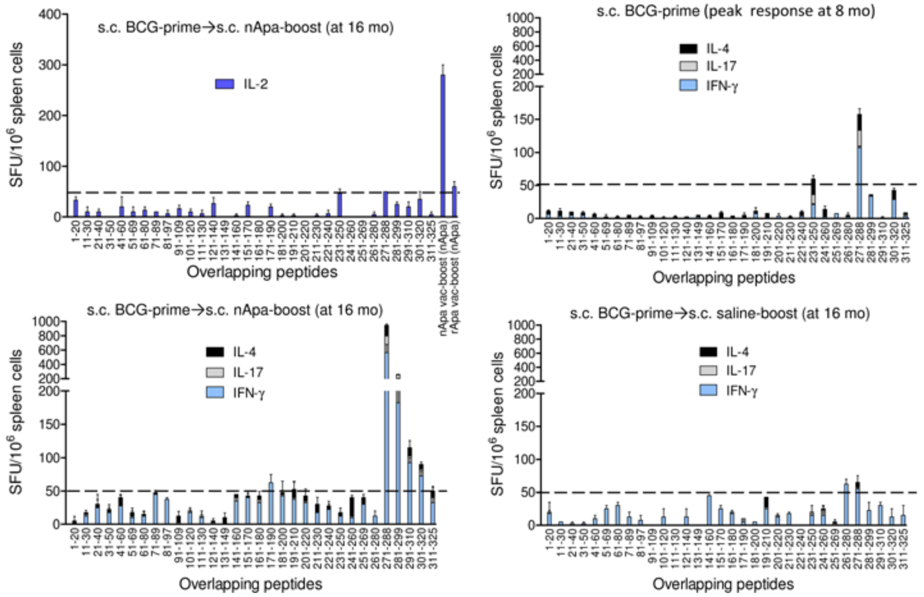

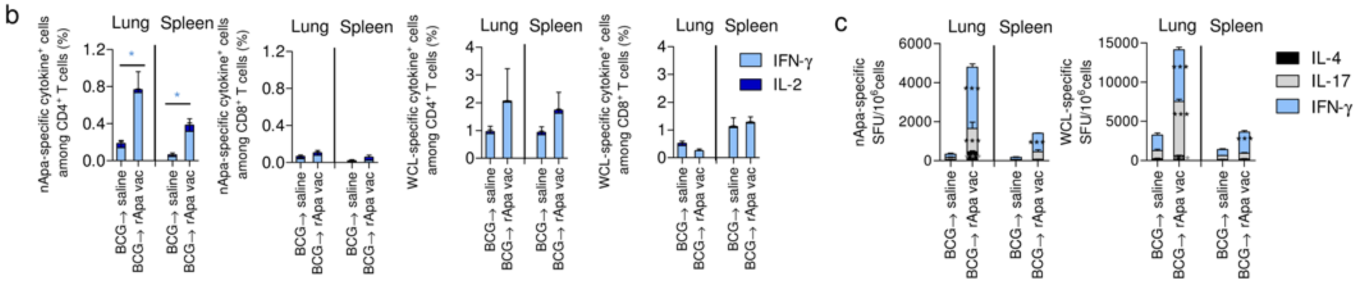

Mice were vaccinated once with BCG using the i.n. or s.c. route. Sixteen months later, mice received two booster doses of nApa or rApa (1µg) in a DDA/MPL adjuvant by a homologous route as depicted in **Figure 5a**. The BCG-primed mice which were left untreated or boosted (× 2) with a saline served as controls. Five weeks after the second boost, mice (n = 4/group) were euthanized and immune responses were investigated. (**a**) Screening of a synthetic overlapping Apa peptides in the s.c. BCG-primed–nApa-boosted and control mice. Pooled splenocytes from indicated groups (n = 4 mice/group) were stimulated *in vitro* with 32 individual synthetic peptides in a cultured ELISPOT assay. Peptide-specific IL-2 (upper left panel) or IFN-γ, IL-17 and IL-4 responses (lower left panel) of the s.c. BCG-primed–nApa-boosted mice at 16 months are plotted as SFU/million cells. The nApa-specific IL-2 response of the s.c. BCG-primed and a subunit nApa- or rApa-boosted mice (upper left panel), the individual peptide-specific IFN-γ, IL-17 and IL-4 responses of the s.c. BCG-primed mice at the peak of response (8 months) (upper right panel) and the s.c. BCG-primed–saline-boosted controls at the 16 months (lower right panel) are shown for comparison. (**b**) Frequencies of nApa- and WCL-specific IFN-γ or IL-2-producing cells among $CD4^+$ and $CD8^+$ T cells in the lung and spleen of a subunit rApa vaccine- or saline-boosted mice using a flow cytometry. Data are of 4-5 individually analyzed mice per group and error bars represent s.e.m. (**c**) Frequencies of nApa- and WCL-specific IFN-γ, IL-17, IL-4 SFU/ million lung or spleen cells of a subunit rApa vaccine- or saline-boosted mice using a cultured ELISPOT. Error bars represent s.d. of triplicate culture. ANOVA followed by Bonferroni's test. **P* < 0.05, ***P* < 0.01 and ****P* < 0.001. s.c., subcutaneous; mo, months; vac, vaccine; WCL, whole cell lysate of *Mtb*; SFU, spot forming units.

Figure S5. T-cell clones generated by a subunit nApa vaccination express an IL-2-producing phenotype.

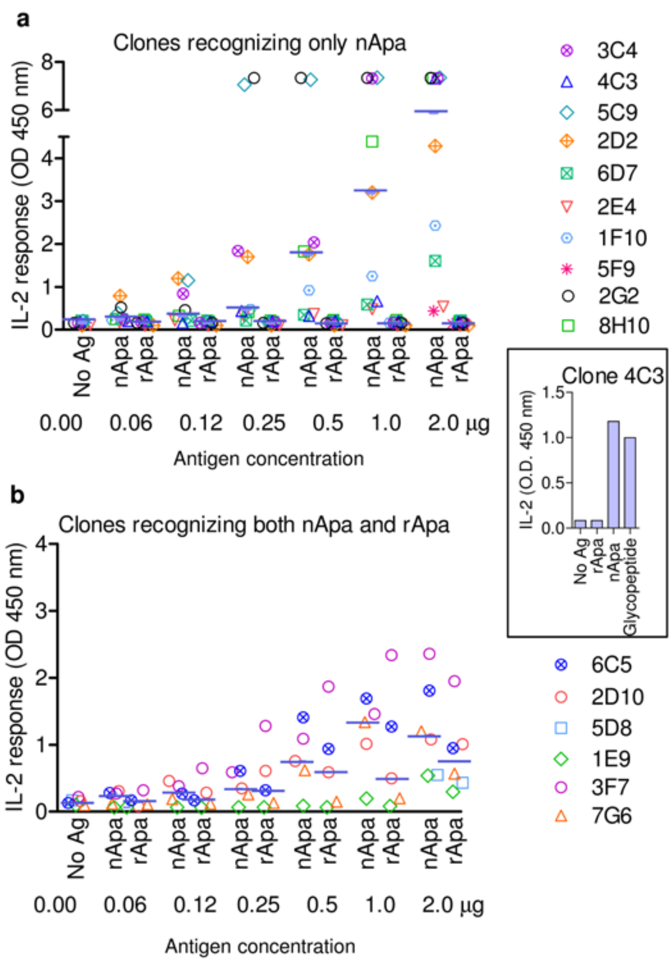

BALB/c mice were vaccinated using a single dose of nApa (40 µg) in the Freund's incomplete adjuvant and the T-cell hybridomas were generated using a draining lymph node cells. Individual T-cell clones responding to nApa were purified by a serial dilution and tested for a reactivity to the native or recombinant form of Apa using an IL-2 capture ELISA, after a co-culture with the syngeneic antigen presenting cells (APCs) pulsed with the antigen. Of the total 17 Apa-reactive hybridoma clones developed, 10 clones recognized only nApa and not rApa (**a**), while 7 clones recognized both nApa and rApa (**b**) and produced a positive IL-2 response. The IL-2 response of one clone that recognized both nApa and rApa but stopped responding consistently is not shown. The antigen dose response curve was

carried out using the antigen-pulsed bone marrow derived dendritic cells as APCs. For clones 5F9 and 5D8 data is available using only one antigen dose (2 µg) per well and BALB/c derived B-cell line A20 as APCs. The horizontal line indicates a median response. None of the 10 nApa-reactive clones recognized any of the synthetic, overlapping, nonmodified peptides of Apa. Of the 10 clones recognizing only nApa, one clone, 4C3, was tested further for the epitope specificity. The T cell clone 4C3 reacted exclusively with the N-terminal *O*-mannosylated glycopeptide fraction (residues p40-145) of trypsin-digested nApa following fractionation by a reversed phase-HPLC column chromatography and produced a positive IL-2 response using 1µg of nApa or glycopeptide fraction (inset). All clones were negative for the IFN-γ production (O.D. 450 < 0.2) testing the same supernatants as in the IL-2 assay, 24 h after *in vitro* stimulation.

Figure S6. Timing and route of a subunit-Apa-boost in the BCG-primed mice influence the magnitude and quality of specific antibody response.

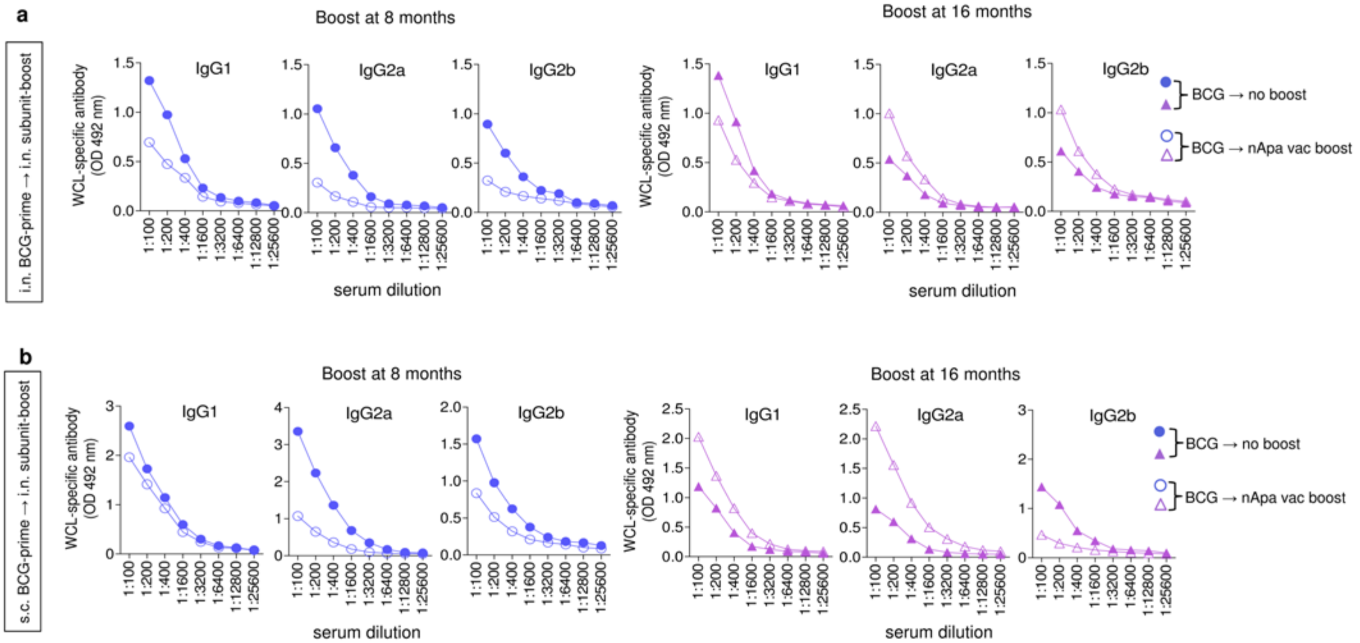

(**a–b**) Serum antibody responses of the BCG-primed and nApa (single 10µg dose)-boosted mice from the experiments using 8 or 16 months of boosting-interval and a homologous i.n.–i.n. or heterologous s.c.–i.n. vaccination routes (**Figure 2a** and **Figure 3a**) were investigated. Because nApa is one of the constituents of WCL, only mycobacteria-specific antibody responses following a subunit-boost were probed. (**a**) Serum IgG responses of the i.n.–i.n. regimen (in **Figure 2)** using ELISA. The WCL-specific IgG1, IgG2a and IgG2b antibody levels are plotted for the nApa-boosted groups (i.e., boosted 8 months or 16 months after BCG-priming) in comparison with the respective BCG-primed non-boosted controls. Regardless of the timing of boost, decreased specific IgG1 antibody levels were found in the sera of nApa-boosted mice relative to levels in the corresponding non-boosted controls. Interestingly, nApa-boost at the contraction-phase (16 months) increased the specific serum IgG2a and IgG2b levels, indicating the influence of the timing of a subunit nApa-boost after BCG-priming on the magnitude and quality of antibody response (**b**) Serum IgG response of the s.c.–i.n. regimen (in **Figure 3)** using ELISA. The WCL-specific IgG1, IgG2a and IgG2b antibody levels are plotted for the nApa-boosted groups (i.e. boosted 8 months or 16 months after BCG-priming) in comparison with the respective BCG-primed non-boosted controls. No increase was found in the serum antibody levels following nApa-boost at the peak of BCG-response (8 months), where higher anti-WCL IgG subclass levels preexisted. On the contrary, nApa-boost during the contraction-phase (16 months) effectively boosted WCL-specific IgG1 and IgG2a but not IgG2b levels, which collectively suggest that the route of prime–boost regimen differentially influences the quality of antibody response. s.c., subcutaneous; i.n., intranasal; vac, vaccine; WCL, whole cell lysate of *Mtb*.

Figure S7. Subunit rApa-boosts by a homologous s.c. route during the contraction-phase maintain specific IL-17-producing cells after *Mtb* challenge and improve waning BCG-induced protection.

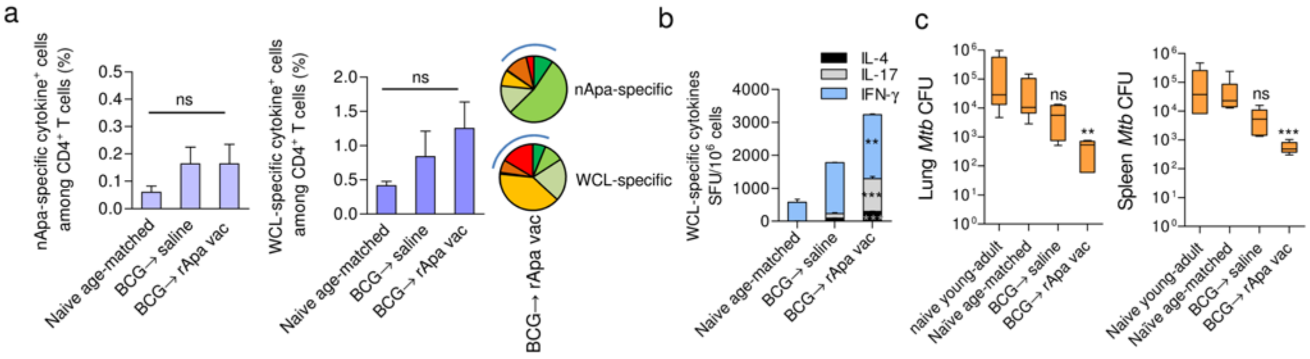

(**a–c**) Mice were prime–boost vaccinated by the s.c. route as depicted in **Figure 5a**. (**a**) Magnitude (histograms) and quality (pie charts) of nApa- and mycobacteria (WCL)-specific total cytokine-producing (IFN-γ, IL-2 and/or TNF-α) cells among splenic $CD4^+$ T cells 6 weeks after *Mtb* challenge using a flow cytometry. Histograms represent mean + s.e.m responses and pie charts show proportions of seven subpopulations of specific cytokine-producing T cells in any combinations constituting the total specific $CD4^+$ T-cell response and present the mean frequencies of seven cytokine subsets (n=4 randomly selected and individually analyzed mice/group). Arcs denote IL-2-producing cells in any combinations. ANOVA followed by Bonferroni's test; ns: non-significant. (**b**) Frequencies of WCL-specific IFN-γ, IL-17 and IL-4 SFU/10^6 splenocytes of mice using a cultured ELISPOT assay. Data are mean + s.d. (n = 4 mice/group). ANOVA followed by Bonferroni's test comparing responses of rApa- and saline-boosted mice. (**c**) Protective efficacy against *Mtb* infection. *Mtb* bacillary-loads in the lung and spleen of rApa vaccine-boosted, saline-boosted, age-matched naïve and young adult naïve (12-week-old) mice 6 weeks post-challenge shown as a box plot (n = 5–6 mice/group). Whiskers indicate maximum and minimum CFU levels. WCL, whole cell lysate of *Mtb*; vac, vaccine; SFU, spot forming units; CFU, colony forming units.

Figure S8. Poor IL-2 response in the BCG-primed–rApa vaccine-boosted mice is not due to the inherent inability of a subunit rApa vaccine to induce specific IL-2^{+}CD4^{+} T cells.

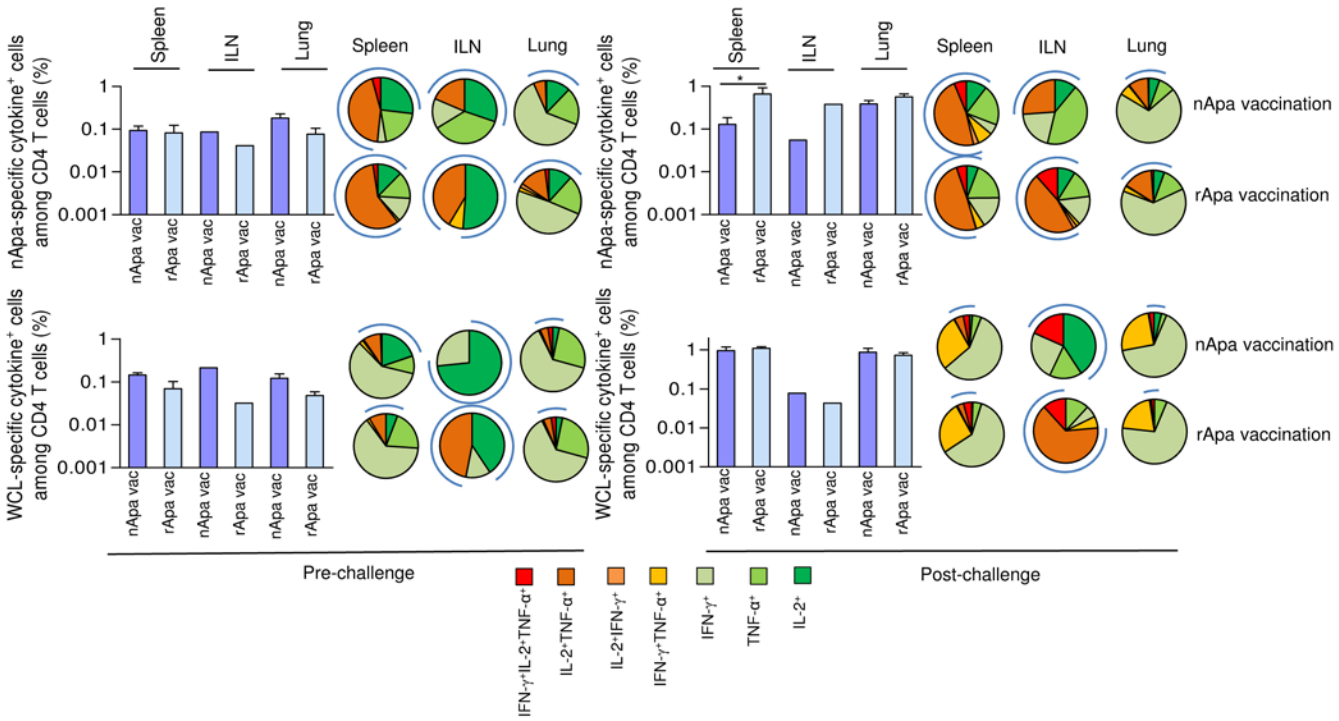

Six-eight-week old mice were vaccinated with 3 doses of nApa or rApa (1µg/dose) in DDA/MPL adjuvant at 4-week intervals by a s.c. route on the hind legs. Magnitude (histograms) and quality (pie charts) of nApa- and WCL-specific total cytokine-producing (IFN-γ, IL-2 and/or TNF-α) cells among CD4^{+} T cells of spleen, ILN and lung were determined 4 weeks after the last subunit dose (pre-challenge) and 6 weeks after *Mtb* challenge (post-challenge) using a flow cytometry. Significant using ANOVA followed by Bonferroni's test. Arcs surrounding pie charts denote IL-2-producing cells in any combinations of nApa- or WCL-specific CD4^{+} T cells (n = 4 individually analyzed mice/group). WCL, whole cell lysate of *Mtb*; vac, vaccine.

Supplementary methods

Vaccinations and experimental infections. *Mtb* H37Rv was grown to mid-log phase at 37° C in liquid glycerol-alanine-salts (GAS) medium and culture filtrate was harvested. The nApa was purified from the culture filtrate by combinations of column chromatography techniques using Con-A-Sepharose and Phenyl-Sepharose columns, while rApa was expressed in *E. coli* BL21 (DE3) and purified from lysates by Nickel chromatography with endotoxin removal step followed by DEAE-Sepharose chromatography.[14,17] Purity was confirmed by SDS-PAGE, N-terminal sequencing and by Western blot using Apa-specific mAb CS-93. The rApa clone pMRLB.17, whole cell lysate (WCL) of *Mtb* H37Rv, rESAT-6, rCFP-10 and mAb CS-93 were obtained through the NIH Biodefense and Emerging Infection Research Resources Repository (BEI resources).

Lyophilized BCG vaccine (Danish 1331 strain, Staten Serum Institute, Copenhagen, Denmark, provided by the Center for Biologics Evaluation and Research, Food and Drug Administration, Bethesda, MD) was suspended in the vaccine diluent (diluted Sauton medium provided by supplier), and the intranasal (i.n.) vaccination was carried out by applying a total of 30 µl of vaccine suspension containing 10^6 BCG CFU drop-wise to the external nares (15 µl per nostril) using a fine tip micropipette and allowing the mouse to inhale the suspension into the lungs naturally.[13] For subcutaneous (s.c.) vaccination, 50 µl of a BCG suspension was injected above the gluteus superficialis and biceps femoralis muscles of both hind legs using a 26-gauge needle to deliver a total of 10^6 CFU. For subunit-boosting, BCG-primed mice received 1–2 doses of either nApa or rApa (1 or 10µg) in DDA-MPL adjuvant (250µg DDA and 25µg MPL-A per dose; Sigma-Aldrich, St. Louis, MO)[13,17] 8 or 16 months after single BCG vaccination as described in the schematics of prime-boost regimens. For subunit vaccination, naïve mice received nApa or rApa (1 or 10µg) in DDA-MPL three times at 2 or 4-week intervals by i.n. (20µl per nostril) or s.c. (100µl per flank) route. Five weeks after final subunit-booster dose, mice were challenged with *Mtb*

Erdman lot K01 (Mycos Research, provided by the Center for Biologics Evaluation and Research, Food and Drug Administration) by airway i.n. route using 5×10^4 CFU Erdman[13,17] in the animal biosafety laboratory (ABSL)-III facilities of the Centers for Disease Control and Prevention (CDC), Atlanta. At 48 h following challenge, about 250 mean CFU could be cultured from the lungs. The *Mtb* bacillary-burden was determined by plating organ homogenates onto Middlebrook 7H10-agar supplemented with OADC (Becton Dickinson, San Diego, CA) and 2-thiophene-carboxylic acid hydrazide (2μg/ml) to selectively inhibit the growth of BCG bacilli in the organs of BCG-primed mice. The CFU were enumerated after 4 weeks of incubation at 37°C.

Collecting blood and tissues and isolation of immune cells. Mice were bled by cardiac route and sacrificed at indicated time points by exsanguination under isoflurane anesthesia. Separated serum samples were stored at -20^0 C until use. The lungs, spleen, inguinal lymph nodes (ILN) and superficial cervical lymph nodes (CLN) were aseptically removed and organ cells were isolated in RPMI-1640 medium supplemented with 100 IU/ml penicillin, 50 μg/ml streptomycin, 1 mM L-glutamine, 25 mM HEPES, 1 mM sodium pyruvate, 5×10^{-5} M β-mercaptoethanol, vitamins and nonessential amino acids (all Gibco-Invitrogen, Grand Island, NY) and 10% endotoxin-tested heat-inactivated FCS (Atlas Biologicals, Fort Collins, CO) as described previously.[13] Briefly, the lungs were digested with an enzyme mixture containing 1 mg/ml collagenase type IV (Sigma-Aldrich) and 25 U/ml DNase (Roche, Penzberg, Germany) in supplemented RPMI-1640 at 37°C for 1 h. The digested lung fragments, spleen or LNs were pressed through a 70-μm pore size cell strainer (BD Falcon, Bedford, MA) to obtain a single cell suspension. The erythrocytes were lysed with RBC lysis buffer (eBioscience, San Diego, CA) at 22°C, and cells were washed extensively (×4), re-suspended in supplemented RPMI-1640 and counted using an automated cell counter (Countess, Invitrogen, Carlsbad, CA) employing the trypan blue dye exclusion method.

Flow-cytometric staining and analysis. Cell surface marker and intracellular cytokine staining (ICS) was performed as described previously.[8,17] Briefly, 2×10^6 cells were dispensed in 96 well polypropylene plates in supplemented RPMI-1640 medium and stimulated with *Mtb* antigen (10µg/ml) in a total volume of 200 µl. Stimulation with PMA-ionomycin (1µg/ml) was used as a positive control. Stimulations were conducted in the presence of anti-CD28 and anti-CD49d antibodies (1µg/ml; BD Pharmingen) and the cells were incubated at 37°C in the presence of 5% CO_2 for 8 h. Brefeldin A (10µg/ml) and monensin (3µM; BD pharmingen) were added and cells were cultured for additional 4 h. Cells were washed in FACS buffer (PBS containing 0.1% sodium azide and 2% FCS) and subsequently surface stained with pre-titrated anti-mouse antibodies against CD3 (Pacific blue-clone 500A2, Phycoerythrin (PE)-clone 500A2, and Allophycocyanin (APC)-clone 145-2C11), CD4 (Alexa Fluor-700-clone RM4-5, PE-clone RM4-5, and Peridinin chlorophyll protein complex (PerCP)-clone RM4-5), CD8 (PerCP-clone 53-6.7 and PE-clone 53-6.7), CD44 (FITC-clone IM7), CD62L (PE-Cy7-clone MEL-14), CCR-7 (Alexa Fluor-488 and PE-clone 4B12), KLRG-1 (APC-clone 2F1) and PD-1 (PE-Cy7-clone RMP1-30 and PE-clone RMP1-30). After permeabilization (Cytofix/Cytoperm kit; BD Pharmingen), intracellular staining was performed using anti-mouse Abs against IFN-γ (APC-clone XMG1.2), TNF-α (PE-Cy7-clone MP6-XT22) and IL-2 (PE-clone JES6-5H4). Cells were washed twice with 1X PermWash and once with FACS buffer and suspended in 1% paraformaldehyde in PBS. Approximately 500,000 lymphocytes were acquired on the LSRII system (BD Immunocytometry Systems) and analyzed using FlowJo software (Treestar, Inc., San Carlos, CA). Lymphocytes were identified based on their scatter patterns, and $CD3^+$, $CD8^-$, $CD4^+$ cells were considered to be CD4 T cells, while $CD3^+$, $CD8^+$, $CD4^-$ cells were considered CD8 T cells. These $CD4^+$ and $CD8^+$ T cells were then gated for cells positive for the respective cytokines. Boolean combination gating was performed to calculate the frequencies of expression profiles corresponding to the seven different combinations of

cytokines by using the FlowJo software. After subtracting the background values, frequencies of individual or total cytokine secreting $CD4^+$ or $CD8^+$ T cells were plotted for each antigen.

ELISPOT assay. IFN-γ, IL-2, IL-4 (BD-Biosciences) or IL-17A (eBioscience) ELISPOT assay was performed using a commercially available mouse reagent set as described previously [8,17] using *Mtb* antigens for *in vitro* stimulations of organ cells at 37°C in the presence of 5% CO_2 for 40 h. In brief, 96-well ELISPOT plates were coated with 100 µl of 5 µg/ml respective capture antibody in PBS (pH 7.2) and incubated overnight at 4°C. Free binding sites were blocked with RPMI-1640 medium containing 10% FCS (Atlas Biologicals) for 2 h at room temperature. Mouse lung, spleen or LN cells were suspended in different dilutions starting at 1 or 2×10^5 cells per well in supplemented RPMI-1640 medium containing 10% FCS. Cells were stimulated with 10 µg/ml of nApa, rApa, WCL, rESAT-6, rCFP-10 or synthetic Apa peptides for 40 h. Stimulation with Con-A (1µg/ml; Sigma-Aldrich) was used as a positive control for cell viability and reactivity. After incubation at 37°C, the wells were washed with PBS-Tween-20 and the site of cytokine secretion was detected with a biotin-labeled detection antibody and horseradish peroxidase-conjugated streptavidin. The enzyme reaction was developed using 3-amino-9-ethylcarbazole (AEC) substrate reagent set (BD-Biosciences, San Diego, CA). The number of SFU per well were counted using an ELISPOT reader (Cellular Technology Limited, Cleveland, OH). The number of spots specific for each antigen preparation was calculated by subtracting the number of spots that formed in the absence of added antigen from the number that formed in its presence. The cytokine response ≥ 50 SFU/10^6 organ cells after subtracting no antigen control values was considered a positive response.

Generation of nApa-specific T-cell hybridomas and cytokine ELISA. T-cell hybridomas specific for *Mtb* nApa were generated as previously described[17]. Briefly, four BALB/c mice were each vaccinated with 40 µg of nApa in FIA by injecting 25 µl into each hind footpad and the remainder at the base of the tail. Five days after the vaccination, the draining lymph nodes (popliteal, inguinal and periaortic) were harvested to obtain LN cells. The primed LN cells were re-stimulated *in vitro* with syngeneic bone marrow derived dendritic cells (BMDCs) that had been pulsed with 10 µg/ml nApa per well. Approximately 1×10^6 primed LN cells were added per well in two 24-well tissue culture plates in a total volume of 1 ml of complete RPMI 1640 medium (RPMI 1640 supplemented with 10% FCS, 5×10^{-5}M 2-ME (Sigma) plus a nutrient cocktail). After two days of culture, the cells were harvested and pooled from all 48 wells, washed, fused with the T cell fusion partner BW$\alpha^-\beta^-$, and plated out into ten 96-well plates. Clones that grew in individual wells were screened using either BMDCs, as above, or a BALB/c mouse-derived B cell lymphoma line A20 pulsed with 0.5µg/well nApa. The selection of responding T cell hybridomas was performed by assaying 24 h culture supernatants using paired rat mAbs specific for mouse IL-2 or IFN-γ (Pharmingen/BD-Biosciences) in a capture ELISA. For antigen presentation studies, micro-culture wells were prepared containing 250 µl of culture medium, 5×10^4 each of T cell hybridoma and antigen presenting cells (APC), and a known amount of nApa or rApa (intact or trypsin digested), in flat-bottomed 96-well micro-titer wells. Dose response curves with native and recombinant antigens were performed to determine which T cell hybridomas respond to nApa alone, or to both native and recombinant form of Apa. T cell hybridomas were further tested with a panel of synthetic, overlapping, nonglycosylated Apa peptides or trypsin digested and RP-HPLC separated nApa fractions[17] to determine the peptide epitopes that were recognized. Antigen-specific IL-2 responses of the clones were comparable regardless of APC type used for antigen presentation (i.e., bone-marrow-derived macrophages (BMDMs) versus BMDCs; data not shown).
